# New species of the genera *Bracon* Fabricius and *Syntomernus* Enderlein (Hymenoptera, Braconidae, Braconinae) from South Korea

**DOI:** 10.3897/zookeys.999.58747

**Published:** 2020-11-30

**Authors:** Konstantin Samartsev, Deok-Seo Ku

**Affiliations:** 1 Zoological Institute, Russian Academy of Sciences, St Petersburg 199034, Russia Zoological Institute, Russian Academy of Sciences St Petersburg Russia; 2 The Science Museum of Natural Enemies, Geochang, 50147, South Korea The Science Museum of Natural Enemies Geochang South Korea

**Keywords:** *
Ficobracon
*, *
Habrobracon
*, new combination, new synonym, *
Osculobracon
*, Palaearctic, taxonomy

## Abstract

Six new species, Bracon (Bracon) kimchanghyoi**sp. nov.**, B. (B.) yeogisanensis**sp. nov.**, B. (Habrobracon) allevatus**sp. nov.**, B. (Osculobracon) perspicillatus**sp. nov.**, *Syntomernus flavus***sp. nov.**, and *S. scabrosus***sp. nov.** are described from South Korea and short keys for their identification are presented. The genus *Ficobracon* van Achterberg & Weiblen, 2000, **syn. nov.** is considered a junior synonym of *Syntomernus* Enderlein, 1920 and new combinations are proposed for *Syntomernus asphondyliae* (Watanabe, 1940), **comb. nov.**, *S. brusi* (van Achterberg & Weiblen, 2000), **comb. nov.**, *S. codonatus* (Huang & van Achterberg, 2013), **comb. nov.**, *S. kashmirensis* (Maqbool, Akbar & Wachkoo, 2018), **comb. nov.**, *S. rhiknosus* (Huang & van Achterberg, 2013), **comb. nov.**, *S. sunosei* (Maeto, 1991), **comb. nov.** (= *Bracon flaccus* Papp, 1996, **syn. nov.**), and *S. tamabae* (Maeto, 1991), **comb. nov.**

## Introduction

With more than 3000 described species, the Braconinae is one of the largest subfamilies of Braconidae (Chen and van Achterberg 2019). As common members of ecosystems, braconines have high species diversity in Palaearctic, but because most of the Palaearctic species belong to the “dustbin” genus *Bracon* Fabricius (Belshaw et al. 2001) their study has been hampered. The Eastern Palaearctic fauna of the subfamily may be more diverse than its European fauna, because being much less studied it already includes a comparable number of known species (Yu et al. 2016).

The fauna of Braconidae of the Korean Peninsula has been intensively investigated (Papp 2009, 2013; Kim et al. 2016; Lee et al. 2016, 2018, 2020a, 2020b; Ku et al. 2020). However, the work on the Braconinae is still complicated. Most of the recent studies on the group were carried out by Jenő Papp (Papp 1996, 1998, 2018) and occasionally by Korean scientists (Ku et al. 2001; Lee et al. 2018; Kang et al. 2019). The present article provides new results based on the extensive Braconinae material collected by the second author in South Korea.

## Materials and methods

### Terminology

Morphological nomenclature follows Quicke (1987) and van Achterberg (1993); the transverse pronotal sulcus is included after Karlsson and Ronquist (2012). Abbreviations of morphological terms:

**Od** maximum diameter of lateral ocellus;

**OOL** ocular-ocellar distance;

**POL** postocellar distance.

Museum acronyms:

**EIHU**Hokkaido University Museum (Sapporo, Japan);

**HNHM**Hungarian Natural History Museum (Budapest, Hungary);

**MIIZ**Museum and Institute of Zoology, Polish Academy of Sciences (Warszawa, Poland);

**MNB**Museum für Naturkunde (Berlin, Germany);

**NIBR**National Institute of Biological Resources (Incheon, South Korea);

**SMNE** Science Museum of Natural Enemies (Geochang, South Korea);

**ZISP**Zoological Institute of the Russian Academy of Sciences (Saint Petersburg, Russia).

### List of collection localities in South Korea (numbers in brackets correspond to the numbers of points on the map in Fig. 1)

**Gangwon-do**: Goseong-gun: [1] Hyeonnae-myeon, Baebong-ri; [2] Hyeonnae-myeon, Machajin-ri; [3] Ganseong-eup: [5] Jinbu-ri; [4] Geojin-eup, Naengcheon-ri, Geonbongsa Temple; [6] Toseong-myeon, Sinpyeong-ri, Seoraksan Mountain (Sinseonbong, or Sinseon-Peaks); Cheorwon-gun, [7] Geunnam-myeon, Yukdan-ri; Inje-gun, [8] Buk-myeon, Yongdae-ri, Seoraksan Mountain, Baekdamsa Temple; Hongcheon-gun, [9] Duchon-myeon, Jangnam-ri (Corn Experimantal Station); Chuncheon-si, [10] Sinbuk-eup, Cheonjeon-ri, Cheonjeon 5-ri; Yeongwol-gun, [11] Kimsatgat-myeon, Nae-ri, Town Gijeon; Taebaek-si: [12] Cheoram-dong: [13] Taebaeksan Mountain.

**Figure 1.**
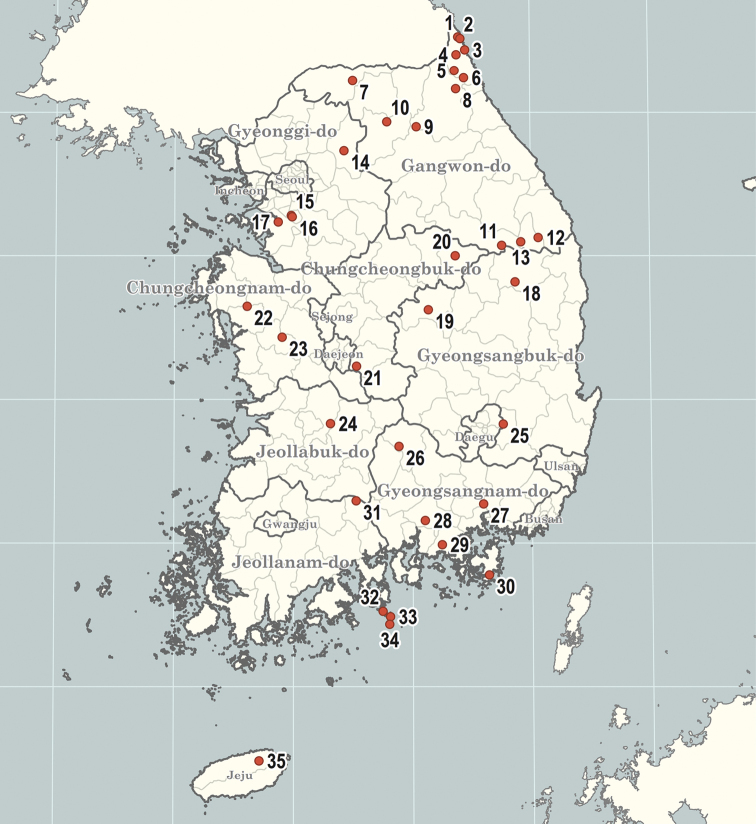
Collecting localities of the material on the described species. Point numbers correspond with numbers in brackets in text.

**Gyeonggi-do**: Gapyeong-gun, [14] Cheongpyeong-myeon, Cheongpyeong-ri, Cheongpyeong Amusement Park; Suwon-si, Gwonseon-gu: [16] Seodun-dong: [15] Yeogisan Mountain; Hwaseong-si, [17] Bibong-myeon.

**Gyeongsangbuk-do**: Bonghwa-gun, [18] Myeongho-myeon, Gwanchang-ri; Mungyeong-si, [19] Buljeong-dong.

**Chungcheongbuk-do**: Danyang-gun, [20] Danyang-eup, Dodam-ri.

**Chungcheongnam-do**: Geumsan-gun, [21] Chubu-myeon, Seongdang-ri, Gaedeoksa Temple; Yesan-gun, [22] Deoksan-myeon, Sudeoksa Temple; Cheongyang-gun, [23] Jeongsan-myeon, Machi-ri.

**Jeollabuk-do**: Jinan-gun, [24] Bugwi-myeon, Sedong-ri, Moraejae Tunnel.

**Gyeongsangbuk-do**: Gyeongsan-si, [25] Yeongnam University.

**Gyeongsangnam-do**: Geochang-gun, [26] Geochang-eup, Songjeong-ri, 35.6712, 127.885; Changwon-si, [27] Uichang-gu, Sogye-dong, Cheonjusan Mountain; Jinju-si, [28] Gajwa-dong; Goseong-gun, [29] Sangni-myeon, Bupo-ri; Geoje-si, [30] Dongbu-myeon, Hakdong-ri.

**Jeollanam-do**: Gurye-gun, [31] Sandong-myeon, Jwasa-ri, Jirisan Mountain (Simwon); Yeosu-si, Nam-myeon: [32] Dumo-ri, Town Moha; [33] Ando Island, Ando-ri; [34] Yeondo Island, Yeondo-ri.

**Jeju-do**: Jeju-si, [35] Jocheon-eup, Seonheul-ri.

The distribution map is generated in R using the packages *sf*, *ggplot2* and *shadowtext* based on the data from https://gadm.org/.

### Material of related species used in diagnoses of taxa and illustrations

**Bracon (Bracon) acunens Papp, 2018**

**Holotype.** South Korea – **Gyeongsangnam-do** • female; Jinju-si, Chojeon-dong [Chojang-dong]; 1 Jul. 1993; D.-S. Ku leg.; at light; 12266/153334; HNHM.

**Other material.** South Korea – **Gyeongsangnam-do** • 1 male; same data as for holotype; 7–8 Jul. 1993; SMNE • 1 female; same data as for holotype; 18–19 Aug. 1993; SMNE • 1 female; Jinju-si, Gajwa-dong; 19 Jun. 1993; D.-S. Ku leg.; SMNE • 1 female; Jinju-si, Naedong-myeon, Doksan-ri; 5–20 May 2003; Tea-Ho Ahn leg.; around the forest road; Malaise trap; SMNE. – **Jeollanam-do** • 1 male; Yeosu-si, Nam-myeon, Ando Island, Ando-ri; 4 Aug. 1993; D.-S. Ku leg.; SMNE.

**Bracon (Bracon) kasparyani Samartsev, 2018**

**Holotype.** Russia – **Primorskiy Kray** • female; Partizansky District, 10 km SE of Partizansk, Novitskoye; 3–4 Aug. 2013; S.A. Belokobylskij leg.; forest, glades; A0065; ZISP.

**Paratypes.** Russia – **Amur Oblast** • 1 female; Arkharinsky District, Khingan Nature Reserve; S.A. Belokobylskij leg.; 17–20 Jul. 2003; forest, forest edges, glades; A0040; ZISP. – **Primorskiy Kray** • 1 male; Khasansky District, env. Lake Khasan, Golubiny Utes; 27 May 1979; S.A. Belokobylskij leg.; forest; A0070; ZISP • 1 female; Nadezhdinsky District, env. Tavrichanka; shrubs; S.A. Belokobylskij leg.; 26 Aug. 1978; A0067; ZISP • 1 female; Nakhodka Urban Okrug, 20 km SW of Nakhodka, Dushkino; S.A. Belokobylskij leg.; 1 Aug. 2013; forest, glades; A0066; ZISP • 1 male; Shkotovsky District, Ussurisky Nature Reserve; 12 Jul. 1973; A.S. Lelej leg.; A0069; ZISP • 1 female; Spassky District, 30 km N of Spassk-Dalny; 4 Sep. 1979; S.A. Belokobylskij leg.; forest; A0006; ZISP • 1 female; Ussuriysk Urban Okrug, env. Ussuriysk, Gornotayozhnaya centre; 2 Aug. 1963; I.M. Kerzhner leg.; A0014; ZISP • 1 female; Vladivostok, Okeanskaya; 11 Aug. 1963; I.M. Kerzhner leg.; A0068; ZISP.

**Bracon (Bracon) kotenkoi Samartsev, 2018**

**Holotype.** Russia – **Primorskiy Kray** • female; Spassky District, Santacheza [now Novoselskoye]; 29 Aug. 1971; Pineker leg.; rice field; sweeping; A0013; ZISP.

**Paratypes.** Russia – **Primorskiy Kray** • 1 female; same data as for holotype; 23 Jul. 1971; A0011; ZISP • 1 female; Spassky District, 20 km SW of Spassk-Dalny, Lake Khanka; 25 Jul. 1998; S.A. Belokobylskij leg.; shore, meadow; A0012; ZISP.

**Bracon (Bracon) kunashiricus Tobias, 2000**

**Holotype.** Russia – **Sakhalin Oblast** • female; Kunashir Island, 6 km N of Mendeleyevo; 4 Aug. 1975; A. Berezantsev leg.; ZISP.

**Bracon (Bracon) sculptithorax Tobias, 2000**

**Holotype.** Russia – **Primorskiy Kray** • female; 80 km NE of Chuguyevka; 28 Jun. 1979; S.A. Belokobylskij leg.; forest; ZISP.

**Bracon (Bracon) sulciferus Tobias, 2000**

**Paratype.** Japan – **Kumamoto Prefecture** • 1 female; Yatsushiro-shi, Izumimachi Momigi; 20 Jul. 1992; V. Makarkin leg.; 700 m; ZISP.

**Bracon (Habrobracon) variegator Spinola, 1808**

**Other material.** Russia – **Saratov Oblast** • 1 male; Krasnokutsky District, near Dyakovka; 14 May 2011; K. Samartsev leg.; fixed sands, shrubs; B0065; ZISP. – **Tyva Republic** • 3 females; env. Uvs Lake; 23–24 Jul. 2009; S.A. Belokobylskij leg.; steppe, flowers; A0101, B0059, B0066; ZISP • 1 female; Tyva Rep., Shara-Nur Lake, 40 km W of Erzin; 26 Jul. 2009; B0060; ZISP – **Volgograd Oblast** • 1 male; Pallasovsky District, Lake Elton, Khara River, Chernyavka area; 15–17 Jun. 2004; S.A. Belokobylskij leg.; steppe, srubs; B0069; ZISP.

Tajikistan – **Khatlon Region** • 1 female, Jilikul, on Vakhsh River; 12 Jun. 1934; V.V. Gussakovskij leg.; B0068; ZISP. – **Region of Republican Subordination** •1 female; Rudaki District, Aruktau Ridge, 15 km W of Gandzhina [= Aktau Ridge?]; 16–17 May 1970; V.I. Tobias leg.; 1800–2000 m; B0067; ZISP.

**Bracon (Osculobracon) cingulator Szépligeti, 1901**

**Holotype.** Russia – **Tatarstan** • female; Kazan; 13 Jun. 1898; E. Csiki leg.; “Exp. Zichy”; 1327/153353; HNHM.

**Bracon (Osculobracon) koreanus Papp, 1998**

**Holotype.** North Korea – **Pyeongannam-do** • female; “Pyong-sung, Bek-sung-li, Za-mo san, 60 km NE from Pyongyan” [Pyeongseong-si, Baeksong-ri, Jamosan Mountain]; 1–10 Aug. 1975; J. Papp and A. Vojnits leg.; 7744/153419; HNHM.

**Other material.** South Korea – **Gyeonggi-do** • 1 female; Paju-si, Gunnae-myeon, Jeomwon-ri; 3 Jun. 1998; Heung-Sik Lee leg.; ZISP. – **Seoul-si** • 1 female; Seongbuk-gu, Anam-dong, Korea University; 1992; D.-S. Ku leg.; ZISP.

**Bracon (Osculobracon) osculator Nees, 1811**

**Other material.** Germany – **Thuringia** • 1 female (lectotype of *B. coniferarum* Fahringer, 1928); Bad Blankenburg; 1898; MNB.

***Syntomernus asphondyliae* (Watanabe, 1940), comb. nov.**

**Paratypes.** Japan – **Tokyo** • 2 females; Hachioji-shi, Takaosan Mountain; emerged 22–23 Sep. 1930; N. Fujita leg.; A0966, A0967; EIHU.

***Syntomernus pusillus* Enderlein, 1920**

**Lectotype.** China – **Taiwan** • female; “Formosa, Takao”; 2 Nov. 1907; H. Sauter leg.; MIIZ.

***Syntomernus sunosei* (Maeto, 1991), comb. nov.**

**Other material.** North Korea – **Hwanghaebuk-do** • 1 female (holotype of *Bracon flaccus* Papp, 1996); “Kaesong, Mts. Pakyon, Pakyon popo, 27 km NE from Kaesong” [13 km NNE of Gaesong, Bakyeonsan = Pakyeon-san Mountain, Bakyeon Pokpo = Pakyeon Falls]; 9 Sep. 1971; S. Horvatovich and J. Papp leg.; 7710/153340; HNHM.

Russia – **Primorskiy Kray** • 1 female; Partizansky District, 10 km SE of Partizansk, Novitsskoe; 3–4 Aug. 2013; S.A. Belokobylskij leg.; forest, glades; A0107; ZISP • 1 female; Spassky District, 20 km SE of Spassk-Dalny, Evseevka; 2 Jul. 2013; S.A. Belokobylskij; forest, forest edges; B0085; ZISP • 1 female; Vladivostok, 10 km SW of Artem; 31 Jul. 2001; S.A. Belokobylskij leg.; forest, forest edges; B0084; ZISP.

***Syntomernus tamabae* (Maeto, 1991), comb. nov.**

**Other material.** Japan – **Hyogo Prefecture** • 2 females; Rokko Mts, Mt. Maya; 5 Nov. 2005; S.A. Belokobylskij leg.; forest; A0139, B0083; ZISP.

## Taxonomy

### Genus *Bracon* Fabricius, 1804

The taxonomic history of the genus has been reviewed by van Achterberg and Polaszek (1996: 25) and Papp (2012: 3); literature summarised in Shenefelt (1978: 1459) and Yu et al. (2016).

#### Bracon (Bracon) kimchanghyoi sp. nov.

Taxon classificationAnimaliaHymenopteraBraconidae

8A63083F-60C0-5893-9F56-A75B69425CD9

http://zoobank.org/5834B7B8-FBD5-4A83-9AD3-9AFB38ACC0E2

Figs 2–9 , 10–18 , 19

##### Type material.

***Holotype.*** South Korea – **Jeollanam-do** • 1 female; Yeosu-si, [34] Nam-myeon, Yeondo Island, Yeondo-ri; 20 Jul. 1993; D.-S. Ku leg.; 324; NIBR.

***Paratypes.*** 5 males. South Korea – **Jeollanam-do** • 3 males; same data as for holotype; 323, 326, 327; SMNE • 1 male; same data as for holotype; 325; ZISP • 1 male; Jeju-si, [35] Jocheon-eup, Seonheul-ri; 26 Aug. 1997; D.-S. Ku leg.; 328; SMNE.

**Figures 2–9.**
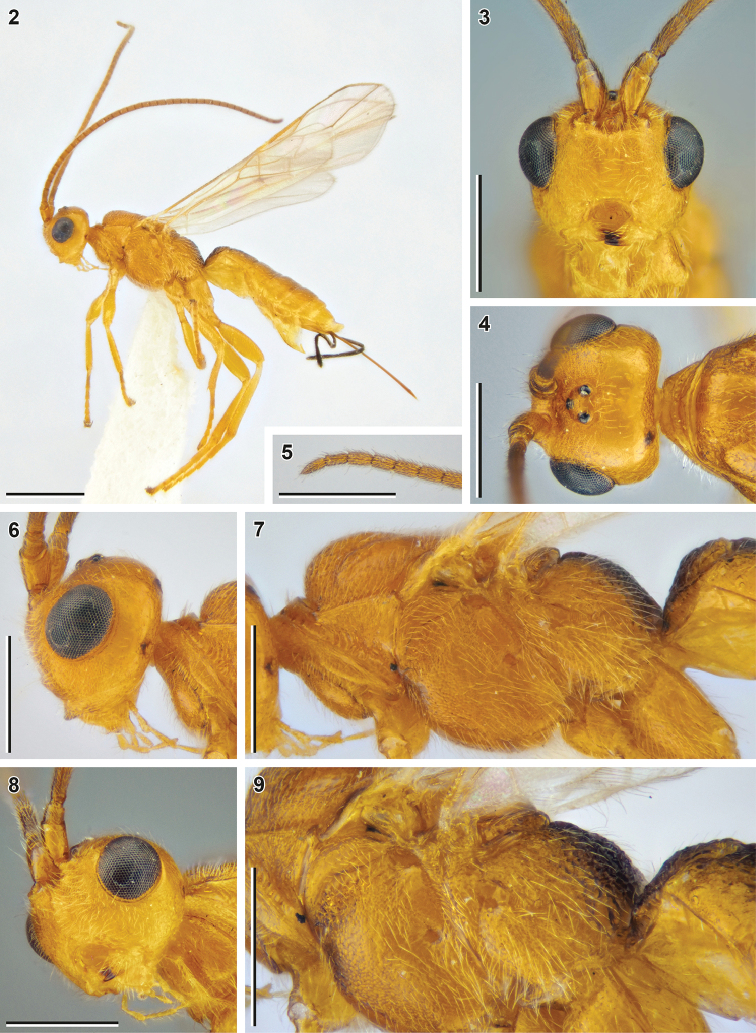
Bracon (Bracon) kimchanghyoi sp. nov. (holotype, NIBR) **2** habitus, lateral view **3** head, front view **4** head, dorsal view **5** Apex of antenna **6** head, lateral view **7** mesosoma, lateral view **8** head, ventrolateral view **9** mesosoma, lateroposterior view. Scale bars: 1 mm (**2**); 0.5 mm (**3–9**).

**Figures 10–18.**
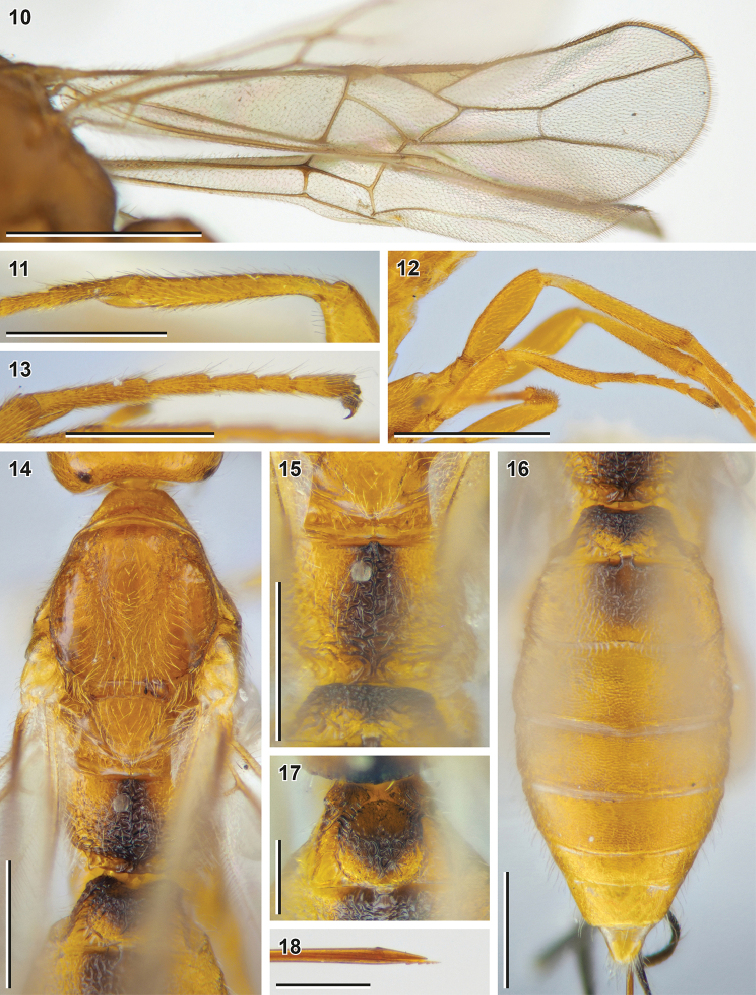
Bracon (Bracon) kimchanghyoi sp. nov. (holotype, NIBR) **10** fore wing **11** fore tibia **12** hind leg, front view **13** hind tarsus **14** mesosoma, dorsal view **15** Propodeum, dorsal view **16** Metasoma, dorsal view **17** first metasomal tergite, dorsal view **18** apex of ovipositor. Scale bars: 1 mm (**10, 12**); 0.5 mm (**11, 13–17**); 0.25 mm (**18**).

##### Etymology.

This species is named in honour of the retired Korean entomologist Prof. Dr. Chang-Hyo Kim.

##### Description.

**Female.** Body length 4.1 mm; fore wing length 3.5 mm.

***Head*.** Width of head (dorsal view) 1.6× its median length. Transverse diameter of eye (dorsal view) 1.4× longer than temple. Eyes with sparse, short setae. OOL 3.8× Od; POL 1.8× Od; OOL 2.1× POL. Frons with deep mid-longitudinal groove. Longitudinal diameter of eye in lateral view 1.3× larger than its transverse diameter. Transverse diameter of eye (lateral view) 1.4× longer than minimum width of temple, hind margins of eye and temple broadened downwards. Face width 1.5× combined height of face and clypeus; 2.2× larger than width of hypoclypeal depression. Longitudinal diameter of eye 1.5× longer than malar space (front view); malar space 1.1× base of mandible. Malar suture absent. Width of hypoclypeal depression 1.1× larger than distance from depression to eye. Clypeus not separated from face by dorsal carina, clypeal sulcus absent, dorsal clypeal margin smoothened. Clypeus flattened, with strongly protruding ventral rim, height of clypeus 0.32× width of hypoclypeal depression. Maxillary palp longer than eye, but shorter than head.

***Antenna*** 1.3× longer than fore wing, with 38 antennomeres. First flagellomere 2.7× longer than its apical width, 1.3× longer than second flagellomere. Middle and penultimate flagellomeres 1.8× and 2.3× longer than wide, respectively. Apical flagellomere pointed.

***Mesosoma*** 1.7× longer than its maximum height. Transverse pronotal sulcus deep and wide, crenulate. Notauli very deep and crenulate anteriorly, impressed and rugulose posteriorly, united near scutellum. Mesoscutum densely setose on notauli, sparsely and widely setose mid-longitudinally. Scutellar sulcus crenulate. Mesepimeral sulcus weakly crenulate, mesopleural pit deep, separated from mesepimeral sulcus. Median area of metanotum (dorsal view) with complete median carina. Metapleural sulcus crenulate. Mid-longitudinal keel on propodeum weak, but complete.

***Wings.*** Fore wing 0.85× as long as body. Pterostigma 4.1× longer than wide. Vein r arising from 0.45 of pterostigma length. Vein 1-R1 1.9× longer than pterostigma. Marginal cell reaching apex of wing. Vein 3-SR 2.8× longer than vein r, 0.50× as long as vein SR1, 1.4× longer than vein 2-SR. Vein 1-M 0.7× vein 1-SR+M, 1.4× vein m-cu, 2.1× longer than vein cu-a. Vein 2-SR+M 0.15× as long as vein 2-SR, 0.20× as long as vein m-cu. Vein 1-CU1 (posterior margin of discal cell) 2.7× longer than vein cu-a. Vein cu-a weakly postfurcal. Vein 2-1A of hind wing absent; vein r-m strongly antefurcal.

***Legs.*** Fore tibia with wide row of long thick setae. Hind femur 3.1× longer than wide. Hind tibia 1.5× longer than hind femur, with subapical transverse row of spiny setae, its inner spur 0.4× as long as hind basitarsus. Hind tarsus as long as hind tibia. Fifth segment (without pretarsus) of hind tarsus 0.5× as long as hind basitarsus and almost as long as second segment. Claws with small almost right-angled basal lobe.

***Metasoma*** 1.3× longer than mesosoma. Median length of first tergite (measured from petiolar tubercle) 0.7× as large as its apical width. First metasomal tergite with developed dorsolateral carinae, incomplete dorsal carinae and distinct mid-longitudinal impression. Median area of first tergite separated by areolate-rugose furrow, 0.6× apical width of tergite. Second tergite medially 0.9× as long as third tergite and 0.7× as large as apical width of first tergite, with weakly impressed s-shaped dorsolateral crenulated impressions. Basal width of second metasomal tergite 1.7× larger than its median length. Suture between second and third tergites deep, weakly curved and crenulate. Apical margins of third to sixth tergites thin, without transverse subapical grooves. Ovipositor sheath 1.4× longer than hind tibia and 0.43× as long as fore wing. Apex of ovipositor with developed dorsal nodus and ventral serration.

***Sculpture.*** Face and frons granulate, vertex weakly granulate, gena weakly coriaceous. Most of mesosoma weakly granulate; metanotum rugose; propodeum anteriorly rugulose, posteriorly rugose, with long transverse rugae along median keel. First metasomal tergite laterally rugulose, its median area weakly rugulose to areolate-rugose; second tergite areolate-rugose to rugose, third–sixth tergites with papillary-like sculpture.

***Colour.*** Body mainly reddish yellow with dark brown patches on propodeum and first and second metasomal tergites. Scape reddish yellow, flagellum yellowish brown. Maxillary palps yellow. Tegulae pale yellow. Wing membrane weakly darkened; pterostigma yellow, wing veins yellowish brown.

**Male.** Body length 2.4–3.8 mm; fore wing length 2.0–3.0 mm.

***Head*.** Width of head (dorsal view) 1.5× its median length. Transverse diameter of eye (dorsal view) 1.3–1.5× longer than temple. OOL 3.5–4.0× Od; POL 1.3–2.4× Od; OOL 1.6–2.6× POL. Longitudinal diameter of eye in lateral view 1.2–1.4× larger than its transverse diameter. Transverse diameter of eye (lateral view) 1.2–1.8× longer than minimum width of temple, hind margins of eye and temple broadened downwards or almost parallel. Face width 1.7–1.9× larger than width of hypoclypeal depression. Longitudinal diameter of eye 1.7–1.8× longer than malar space (front view); malar space 0.85–1.05× base of mandible. Width of hypoclypeal depression 1.1–1.4× larger than distance from depression to eye. Dorsal clypeal margin sharp.

***Antenna*** 1.4–1.5× longer than fore wing, with 29–36 antennomeres. First flagellomere 2.6–3.0× longer than its apical width, 1.1–1.2× longer than second flagellomere. Middle and penultimate flagellomeres 1.9–2.6× and 2.0–2.8× longer than wide, respectively.

***Mesosoma*** 1.8–2.0× longer than its maximum height. Median lobe of mesoscutum some× widely glabrous anteromedially.

***Wings.*** Pterostigma 4.4–4.8× longer than wide. Vein r arising from 0.45–0.50 of pterostigma length. Vein 1-R1 1.8–1.9× longer than pterostigma. Vein 3-SR 1.9–3.1× longer than vein r, 0.45–0.60× as long as vein SR1, 1.1–1.7× longer than vein 2-SR. Vein 1-M 0.7–0.8× vein 1-SR+M, 1.7× vein m-cu. 2.4–3.1× longer than vein cu-a. Vein 2-SR+M 0.10–0.15× as long as vein 2-SR, 0.20–0.25× as long as vein m-cu. Vein 1-CU1 (posterior margin of discal cell) 2.6–3.4× longer than vein cu-a. Vein cu-a weakly postfurcal.

***Legs.*** Hind femur 3.4–4.3× longer than wide. Inner spur of hind tibia 0.3–0.4× as long as hind basitarsus. Fifth segment (without pretarsus) of hind tarsus 0.48–0.53× as long as hind basitarsus and 0.85–0.95× as long as second segment.

***Metasoma*** 1.5–1.7× longer than mesosoma. Median length of first tergite (measured from petiolar tubercle) 0.9–1.0× as large as its apical width. Second tergite medially as long as third tergite and 1.0–1.2× larger than apical width of first tergite. Basal width of second tergite 1.1–1.2× larger than its median length.

##### Diagnosis.

*Bracon kimchanghyoi* sp. nov. is very similar to recently described *B. kotenkoi* Samartsev, 2018, which also has an elongate body, the long malar space, the widely sculptured propodeum and more or less completely sculptured metasoma. The differences between two species are presented in the dichotomy below.

**Table:** 

1	Vertex, propleuron, scutellum and gena smooth (Figs 20, 21, 23); mesoscutum smooth with weak rugulosity along notauli; mesopleuron weakly coriaceous to smooth. Median area of metanotum (dorsal view; Fig. 21) with incomplete median carina (not crossing posterior elevation). Mid-longitudinal keel on propodeum mostly absent, distinct only basally and apically (Fig. 21). Median lobe of mesoscutum anteromedially glabrous (in females; Figs 20, 23; males unknown). Vein 2-SR+M 0.45–0.55× as long as vein m-cu (Fig. 22). Vein 3-SR 3.4–3.6× longer than vein r. Vein 1-R1 1.5–1.6× longer than pterostigma. Tarsal claws with rounded, not protruding basal lobes (Fig. 24). Antenna 0.82–0.96× as long as fore wing	**Bracon (Bracon) kotenkoi Samartsev, 2018**
–	Vertex, propleuron, mesoscutum, scutellum and mesopleuron widely and weakly granulate (Fig. 4, 7, 14); gena weakly coriaceous (Fig. 6). Median area of metanotum (dorsal view; Fig. 15) with complete median carina. Mid-longitudinal keel on propodeum complete (Fig. 15, 19). Median lobe of mesoscutum anteromedially sparsely and widely setose (in females; Fig. 14). Vein 2-SR+M 0.20× (males: 0.20–0.25×) as long as vein m-cu (Fig. 10). Vein 3-SR 2.8× (males: 1.9–3.1×) longer than vein r. Vein 1-R1 1.9× (males: 1.8–1.9×) longer than pterostigma. Tarsal claws with rectangular, somewhat protruding basal lobes (Fig. 13). Antenna 1.3× (males: 1.4–1.5×) longer than fore wing	**B. (B.) kimchanghyoi sp. nov.**

**Figures 19–24.**
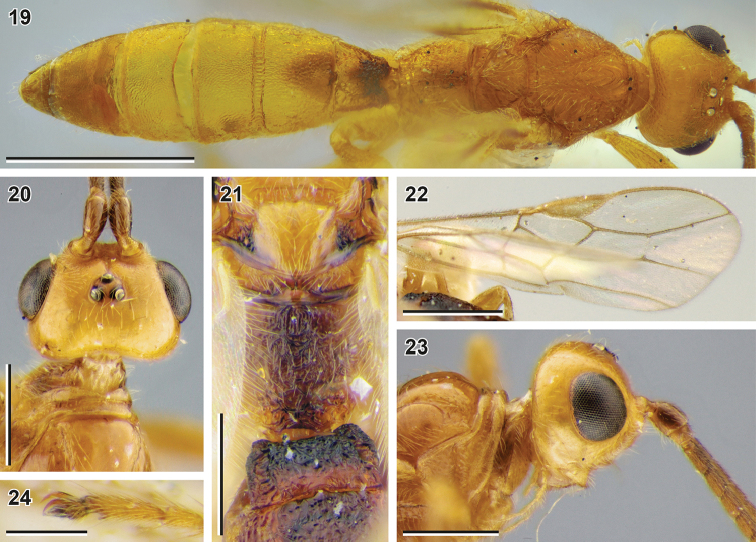
Bracon (Bracon) kimchanghyoi sp. nov. (**19** male paratype, NIBR) and *Bracon kotenkoi* Samartsev, 2018 (**20–24** holotype, ZISP) **19** body, dorsal view **20** head, dorsal view **21** propodeum, dorsal view **22** fore wing **23** head, lateral view **24** apex of hind tarsus. Scale bars: 1 mm (**19, 22**), 0.5 mm (**20, 21, 23**); 0.25 mm (**24**).

#### Bracon (Bracon) yeogisanensis sp. nov.

Taxon classificationAnimaliaHymenopteraBraconidae

28751CC0-2605-565E-988D-0FF579776DF6

http://zoobank.org/B63522FE-BBD6-482B-92BC-7544BFF88248

Figs 25–32 , 33–40

##### Type material.

***Holotype.*** South Korea – **Gyeonggi-do** • female; Suwon-si, [15] Gwonseon-gu, Seodun-dong, Yeogisan Mountain; 11 May 1994; D.-S. Ku leg.; 867; NIBR.

***Paratypes.*** (21 females, 9 males). South Korea – **Gangwon-do** • 1 female; Goseong-gun, [6] Toseong-myeon, Sinpyeong-ri, Seoraksan Mountain; 2 Aug. 2002–19 Oct. 2002; D.-S. Ku leg.; Malaise trap; 888; SMNE. – **Gyeonggi-do** • 4 males; same data as for holotype; 22 Apr. 1994; 891, 892, 893, 895; SMNE • 1 female; same data as for holotype; 29 Apr. 1994; 874; ZISP • 4 females; same data as for holotype; 864, 866, 868, 873; SMNE • 3 males; same data as for holotype; 869, 871, 872; SMNE • 1 male; same data as for holotype; 870; ZISP • 1 female; same data as for holotype; 11–19 May 1994; 879; SMNE • 1 female; same data as for holotype; 19–26 May 1994; 878; SMNE • 1 female; same data as for holotype; 27 May 1996; June-Yeol Choi leg.; Malaise trap; 882; SMNE • 1 female; same data as for holotype; 29 May–6 Jul. 1994; Malaise trap; 877; SMNE • 1 female; same data as for holotype; 16–23 Jun. 1994; 889; SMNE • 1 female; same data as for holotype; 23–29 Jun. 1994; Malaise trap; 890; SMNE • 2 females; same data as for holotype; 10 Jul. 1995; June-Yeol Choi leg.; Malaise trap; 885, 886; SMNE • 1 female; same data as for preceding; 887; ZISP • 1 female; same data as for preceding; 11 Jul. 1997; 884; SMNE • 1 female; same data as for preceding; 14 Aug. 1995; 883; SMNE • 2 females; Suwon-si, [16] Gwonseon-gu, Seodun-dong; 3–11 May 1994; D.-S. Ku leg.; 875, 876; SMNE • 1 male; same data as for preceding; 15 Jun. 1994; 894; SMNE • 1 female; Hwaseong-si, [17] Bibong-myeon; 1 Jun. 1994; D.-S. Ku leg.; 880; SMNE. – **Jeollabuk-do** • 1 female; Jinan-gun, [24] Bugwi-myeon, Sedong-ri, Moraejae Tunnel; 16 Jun. 1996; D.-S. Ku leg.; 881; SMNE.

**Figures 25–32.**
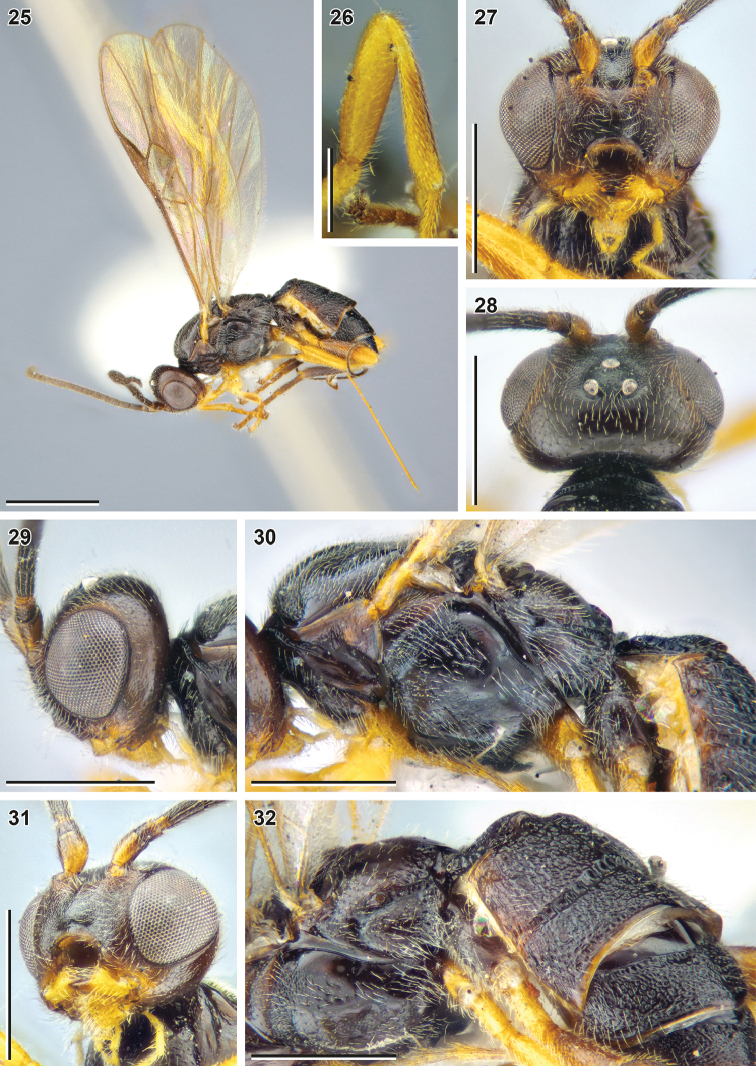
Bracon (Bracon) yeogisanensis sp. nov. (holotype, NIBR) **25** habitus, lateral view **26** fore femur and tibia, front view **27** head, front view **28** head, dorsal view **29** head, lateral view **30** mesosoma, lateral view **31** head, ventrolateral view **32** mesosoma, lateroposterior view. Scale bars: 1 mm (**25**); 0.5 mm (**27–32**); 0.25 mm (**26**).

**Figures 33–40.**
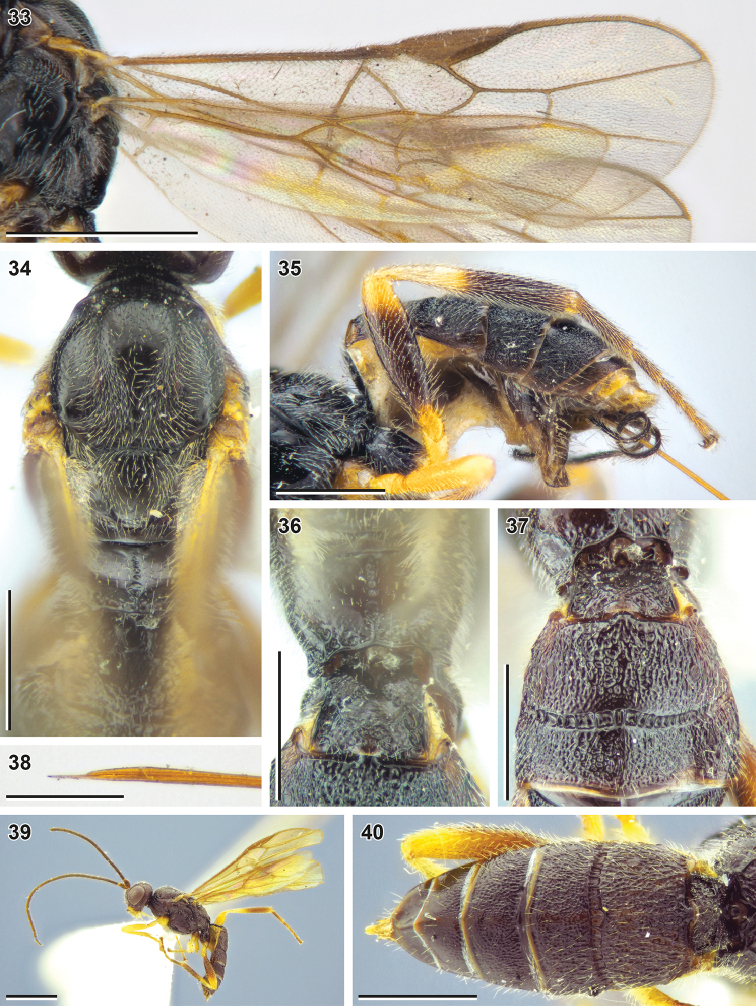
Bracon (Bracon) yeogisanensis sp. nov. (**33–37** holotype, NIBR, **19, 40** male paratype, SMNE) **33** wings **34** mesosoma, dorsal view **35** hind leg, front view **36** propodeum and first metasomal tergite, dorsal view **37** Second and third metasomal tergites, dorsal view **38** apex of ovipositor, lateral view **39** habitus, lateral view **40** metasoma, dorsal view. Scale bars: 1 mm (**33, 39**); 0.5 mm (**34–37, 40**); 0.25 mm (**38**).

##### Etymology.

The name refers to Yeogisan Mountain, the type locality of the species.

##### Description.

**Female.** Body length 2.5–3.1 mm; fore wing length 2.9–3.3 mm.

***Head*.** Width of head (dorsal view) 1.7–1.9× its median length. Transverse diameter of eye (dorsal view) 1.7–1.9× longer than temple. Eyes with sparse, short setae. OOL 2.2–2.5× Od; POL 1.2–1.5× Od; OOL 1.6–1.9× POL. Frons with weak mid-longitudinal groove. Longitudinal diameter of eye in lateral view 1.3× larger than its transverse diameter. Transverse diameter of eye (lateral view) 2.1–2.3× longer than minimum width of temple, hind margins of eye and temple parallel or slightly broadened downwards or upwards. Face width 1.6–1.8× combined height of face and clypeus; 1.8–2.2× larger than width of hypoclypeal depression. Longitudinal diameter of eye 3.2–3.5× longer than malar space (front view); malar space 0.6–0.7× base of mandible. Malar suture absent. Width of hypoclypeal depression 1.5–1.9× larger than distance from depression to eye. Clypeus not separated from face by dorsal carina, clypeal sulcus impressed, dorsal clypeal margin sharp. Clypeus prominent, with protruding ventral rim, height of clypeus 0.2–0.3× width of hypoclypeal depression. Maxillary palp longer than eye, but shorter than head.

***Antenna*** 0.77–0.82× as long as fore wing, with 23–25 antennomeres. First flagellomere 2.1–2.9× longer than its apical width, 1.1–1.3× longer than second flagellomere. Middle and penultimate flagellomeres 1.7–2.2× and 1.5–2.0× longer than wide, respectively. Apical flagellomere spiculate.

***Mesosoma*** 1.5–1.6× longer than its maximum height. Transverse pronotal sulcus deep and anteriorly crenulate. Notauli very deep and crenulate anteriorly, shallowly impressed and smooth posteriorly. Mesoscutum widely setose mid-longitudinally, on notauli and laterally and evenly setose medioposteriorly. Scutellar sulcus crenulate, mesepimeral sulcus weakly crenulate, metapleural sulcus crenulate. Mesopleural pit small or weakly impressed and separated from mesepimeral sulcus. Median area of metanotum (dorsal view) with incomplete median carina. Propodeum with short and branching mid-longitudinal keel apically and weakly foveate or crenulated mid-longitudinal impression in basal half.

***Wings.*** Fore wing 1.1–1.2× longer than body. Pterostigma 2.3–2.8× longer than wide. Vein r arising from basal 0.35–0.45 of pterostigma length. Vein 1-R1 1.7–1.8× longer than pterostigma. Marginal cell ca. 10–20× longer than distance from its apex to apex of wing. Vein 3-SR 1.7–2.0× longer than vein r, 0.42–0.46× as long as vein SR1, 1.1× longer than vein 2-SR. Vein 1-M 0.74–0.77× vein 1-SR+M, 2.3–2.6× vein m-cu. 2.0–2.4× longer than vein cu-a. Vein 2-SR+M 0.08–0.16× as long as vein 2-SR, 0.20–0.45× as long as vein m-cu. Vein 1-CU1 (posterior margin of discal cell) 2.4–2.7× longer than vein cu-a. Vein cu-a interstitial or weakly postfurcal. Vein 2-1A of hind wing absent or very short; vein r-m antefurcal.

***Legs.*** Fore tibia with wide row of long thick setae and transverse apical row of thick setae. Hind femur 3.0–3.8× longer than wide. Hind tibia without subapical row of thick setae, 1.4–1.5× longer than hind femur, its inner spur 0.35–0.37× as long as hind basitarsus. Hind tarsus almost as long as hind tibia, its fifth segment (without pretarsus) 0.37–0.43× as long as hind basitarsus and 0.75–0.80× as long as second segment. Claws with acute angularly protruding basal lobe.

***Metasoma*** 1.0–1.2× longer than mesosoma. Median length of first tergite (measured from petiolar tubercle) 0.7–0.8× as large as its apical width. Dorsolateral carinae of first metasomal tergite developed; dorsal carinae incomplete and weakly curved toward apex of tergite. Median area of first tergite separated by rugose furrow, 0.6–0.7× apical width of tergite. Second tergite medially 1.00–1.15× as long as third tergite and 0.7–0.9× as large as apical width of first tergite, with shallow s-shaped dorsolateral crenulated impressions. Basal width of second metasomal tergite 1.4–1.6× larger than its median length. Suture between second and third tergites deep and wide, curved and crenulate. Apical margins of third to sixth tergites thick, with deep crenulate transverse subapical grooves. Ovipositor sheath 1.2–1.4× longer than hind tibia and 0.33–0.47× as long as fore wing. Apex of ovipositor with weak nodus and weak ventral serration.

***Sculpture.*** Face and frons granulate. Gena and anterior half of vertex coriaceous. Mesopleuron almost smooth, weakly coriaceous or weakly granulate. Mesoscutum medio-posteriorly weakly granulate. Scutellum and metanotum smooth. Propodeum hardly coriaceous to smooth, with short rugae apically. First metasomal tergite laterally and posteriorly rugose; second to fifth tergites areolate-rugose or foveolate-rugose to rugulose-punctate or irregularly punctate; sixth tergite weakly irregularly punctate or almost smooth.

***Colour.*** Body mainly dark brown. Most of scape, mandible, tegulae, fore and middle legs, trochanter and apex of femur of hind leg brownish yellow or yellowish brown. Maxillary palp and base of hind tibia pale yellow. Lateral margins of second and third metasomal tergite and seventh tergite brown or yellowish brown. Wing membrane brownish darkened; pterostigma brown, basally with small pale brown patch; wing veins brown.

**Male.** Body length 2.5–3.2 mm; fore wing length 2.5–3.1 mm. OOL 1.8–2.0× Od, 1.3–1.5× POL. Hind margins of eye and temple broadened upwards (lateral view). Longitudinal diameter of eye 3.9× longer than malar space (front view). Mid-longitudinal keel developed in apical third of propodeum. Vein r-m of hind wing interstitial. Fifth segment (without pretarsus) of hind tarsus ca. 0.9× as long as second segment. Median length of first tergite (measured from petiolar tubercle) 0.90–0.95× as large as its apical width. Second tergite medially 1.1× as large as apical width of first tergite. Basal width of second metasomal tergite 1.3–1.4× larger than its median length. Apical metasomal segments as dark as proximal segments. Otherwise similar to female.

##### Diagnosis.

The new species belongs to a distinct species group including five species known from the Russian Far East, the Korean Peninsula and Japan (*Bracon acunens* Papp, 2018, *B. kunashiricus* Tobias, 2000, *B. sculptithorax* Tobias, 2000, *B. sulciferus* Tobias, 2000, and *B. yeogisanensis* sp. nov.). The species share the following common characters: malar suture absent; face and frons granulate; gena, vertex, mesopleuron and mesoscutum partly with weak granulate or coriaceous sculpture; mesosoma elongate, 1.5–1.7× longer than its maximum height; mesoscutum widely setose medially; notauli deep anteriorly and shallow posteriorly; precoxal sulcus vaguely or shallowly impressed; propodeum with crenulated or foveate mid-longitudinal impression in basal half and with branching mid-longitudinal keel in its apical half; marginal cell of fore wing not shortened, 6–25× longer than distance from its apex to apex of wing; vein r arising distinctly before middle of pterostigma; vein 1-SR+M more or less curved anteriorly; vein cu-a interstitial or weakly postfurcal; wing membrane weakly brownish darkened; coxae without granulate sculpture; hind tibia without transverse apical row of thick setae apically; second segment of hind tarsus 1.1–1.3× longer than fifth segment; claws with acute basal lobes; dorsolateral carinae of first metasomal tergite developed; median area of second tergite absent or very short and weak; dorsolateral s-shaped impressions of second tergite more or less distinct; suture between second and third tergites deep and crenulate; apical margins of third to sixth tergites thick; metasoma completely sculptured (areolate-rugose to irregularly punctate); ovipositor sheath 1.0–1.5× as long as hind tibia, 0.3–0.5× as long as fore wing. Differences between these species are listed in the key below.

**Table:** 

1	Propodeum entirely rugose (Fig. 53). Face weakly, but widely elevated medially (Figs 41, 49), its width 1.4× combined height of face and clypeus. Longitudinal diameter of eye 2.0× longer than malar space (front view; Fig. 41); malar space 1.2× base of mandible. Hind coxa dorsally rugose (Fig. 53). Maxillary palp shorter than eye, brownish yellow. Flagellum bicolored, rusty brown, becoming darker apically (Figs 41, 49). Hind femur 4.6× longer than wide. Ovipositor sheath almost as long as hind tibia. Vein 3-SR 2.5× longer than vein r. Scape brown	**Bracon (Bracon) sculptithorax Tobias, 2000**
–	Propodeum at least anteriorly widely smooth (Figs 36, 54–56). Face not elevated medially (Figs 27, 42–44), its width 1.6–1.8× combined height of face and clypeus. Longitudinal diameter of eye more than 2.5× longer than malar space (front view); malar space 0.55–0.75× base of mandible. Hind coxa smooth (Figs 32, 35). Maxillary palp as long or longer than eye, (pale) yellow. Flagellum uni-coloured, brown or reddish brown	**2**
2	Suture between second and third metasomal tergites almost straight (Fig. 62). Apical margins of third to sixth metasomal tergites not separated by transverse subapical grooves. Fifth metasomal tergite foveolate-rugose. Scape darker, reddish brown (Fig. 42)	**B. (B.) kunashiricus Tobias, 2000**
–	Suture between second and third metasomal tergites more or less curved (Figs 37, 63, 64). Apical margins of third to sixth tergites with deep crenulate transverse subapical grooves. Fifth metasomal tergite without foveolate sculpture, shagreen, irregularly punctate, rugulose-punctate or (in *B. acunens*) areolate-rugose. Scape lighter-coloured, reddish yellow or brownish yellow, often laterally brown (Figs 31, 43, 52)	**3**
3	Hind margins of eye and temple (in lateral view) weakly broadened downwards (Fig. 51). Second metasomal tergite longitudinally rugose (Fig. 63). Suture between second and third tergites weakly curved. Pterostigma without small pale brown patch basally. Vein cu-a postfurcal (Fig. 67). Longitudinal diameter of eye 3.0× longer than malar space (front view; Fig. 43). Transverse diameter of eye (lateral view) 2.7× longer than minimum width of temple. Hind basitarsus 1.9× longer than second tarsal segment, 2.4× longer than fifth tarsal segment. Second metasomal tergite with weak dorsolateral impressions (Fig. 63). Basal width of second metasomal tergite 1.5× larger than its median length	**B. (B.) sulciferus Tobias, 2000**
–	Hind margins of eye and temple (in lateral view) almost parallel (Figs 29, 52). Suture between second and third tergites curved (Figs 37, 64). Second metasomal tergite without longitudinal rugosity. Pterostigma with small pale brown patch basally (Fig. 68). Vein cu-a (almost) interstitial	**4**
4	Second metasomal tergite laterally pale yellow, face and mesoscutum with rusty-brown pattern (Figs 44, 48, 60, 64). Dorsolateral impressions of second metasomal tergite deep (Fig. 64). Hind basitarsus 1.6× longer than second tarsal segment, 1.9–2.1× longer than fifth segment. Basal width of second metasomal tergite 1.7–1.9× larger than its median length. Longitudinal diameter of eye 3.5–3.6× (males: 4.2–4.4×) longer than malar space (front view; Fig. 44). Transverse diameter of eye (lateral view) 2.4–2.5× (males: 2.8–2.9×) longer than minimum width of temple (Fig. 52)	**B. (B.) acunens Papp, 2018**
–	Head, mesosoma and metasoma evenly dark-brown or brownish black (Figs 27, 30, 34). Dorsolateral impressions of second metasomal tergite almost indistinct (Figs 37, 40). Hind basitarsus 1.8–2.1× longer than second tarsal segment, 2.3–2.7× longer than fifth segment (Fig. 35). Basal width of second metasomal tergite 1.4–1.6× larger than its median length (Fig. 37). Longitudinal diameter of eye 3.2–3.5× (males: 3.9×) longer than malar space (front view; Fig. 27). Transverse diameter of eye (lateral view) 2.1–2.3× (males: 2.3–2.4×) longer than minimum width of temple (Figs 29, 39)	**B. (B.) yeogisanensis sp. nov.**

**Figures 41–56.**
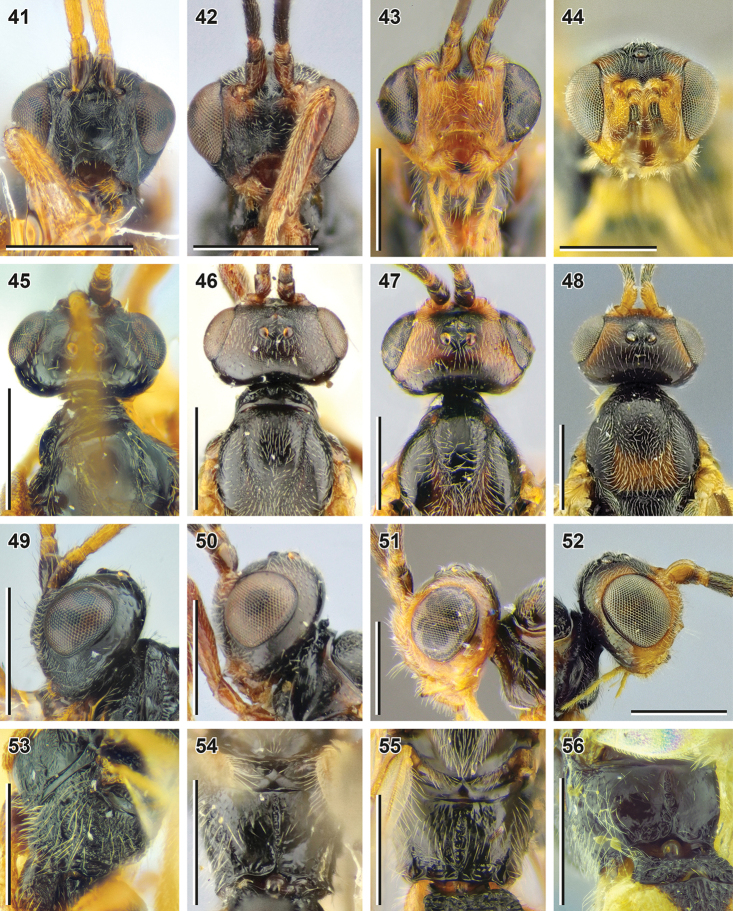
*Bracon sculptithorax* Tobias, 2000 (**41, 45, 49, 53** holotype, ZISP), *B. kunashiricus* Tobias, 2000 (**42, 46, 50, 54** holotype, ZISP), *B. sulciferus* Tobias, 2000 (**43, 47, 51, 55** paratype, ZISP) and *B. acunens* Papp, 2018 (**44, 48, 52** holotype, HNHM**56** female, SMNE) **41–44** head, front view **45–48** head and mesoscutum, dorsal view **49–52** head, lateral view **53–56** propodeum, dorsal view. Scale bars: 0.5 mm.

**Figures 57–68.**
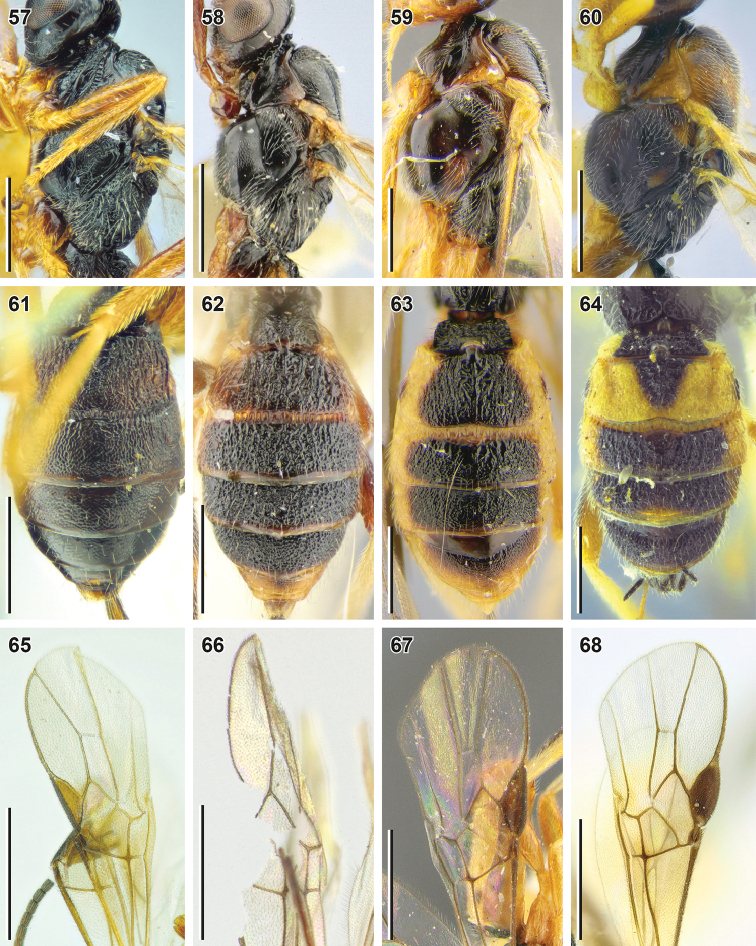
*Bracon sculptithorax* Tobias, 2000 (**57, 61, 65** holotype, ZISP), *B. kunashiricus* Tobias, 2000 (**58, 62, 66** holotype, ZISP), *B. sulciferus* Tobias, 2000 (**59, 63, 67** paratype, ZISP) and *B. acunens* Papp, 2018 (**60, 64** female, SMNE**68** holotype, HNHM) **57–60** mesosoma, lateral view **51–64** metasoma, dorsal view **65–68** fore wing apex. Scale bars: 0.5 mm (**57–64**); 1 mm (**65–68**).

#### Bracon (Habrobracon) allevatus sp. nov.

Taxon classificationAnimaliaHymenopteraBraconidae

64A19C2C-5E35-5254-8473-B7323003F008

http://zoobank.org/CD0B26C9-A677-45B0-9C10-74682E9D0929

Figs 69–78 , 79–85

##### Type material.

***Holotype.*** South Korea – **Jeollanam-do** • female; Yeosu-si, [33] Nam-myeon, Ando Island, Ando-ri; 4 Aug. 1993; D.-S. Ku leg.; 629; NIBR.

**Figures 69–78.**
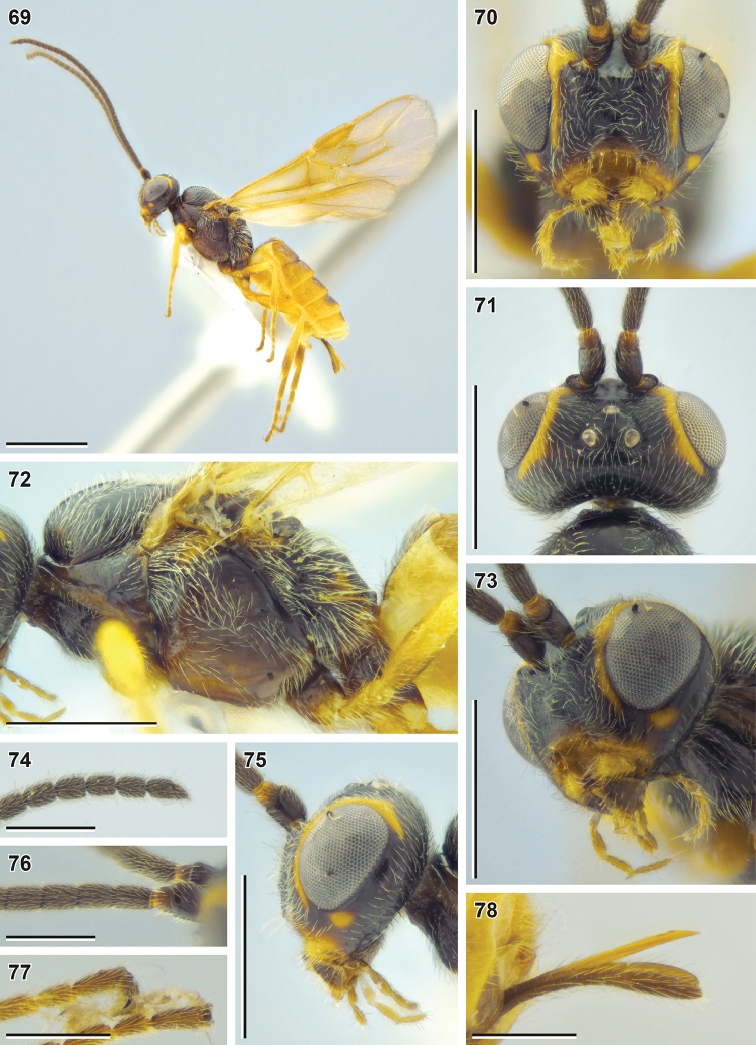
Bracon (Habrobracon) allevatus sp. nov. (holotype, NIBR) **69** habitus, lateral view **70** head, front view **71** head, dorsal view **72** mesosoma, lateral view **73** head, ventrolateral view **74** apex of antenna **75** head, lateral view **76** base of antenna **77** apex of hind tarsus **78** ovipositor. Scale bars: 1 mm (**69**); 0.5 mm (**70–73, 75**); 0.25 mm (**74, 76–78**).

***Paratypes.*** 23 females, 14 males. South Korea – **Gangwon-do** • 2 females; Goseong-gun, [1] Hyeonnae-myeon, Baebong-ri; 26 May 1993; D.-S. Ku leg.; 632, 634; SMNE • 1 male; same data as for preceding; 633; SMNE • 1 male; Goseong-gun, [2] Hyeonnae-myeon, Machajin-ri; 25 May 1993; D.-S. Ku leg.; 661; SMNE • 1 male; Goseong-gun, [3] Ganseong-eup; 25 May 1993; D.-S. Ku leg.; 635; SMNE • 2 females; Goseong-gun, [4] Geojin-eup, Naengcheon-ri, Geonbongsa Temple; 25 May 1993; D.-S. Ku leg.; 637, 638; SMNE • 1 female; same data as for preceding; 639; ZISP • 1 male; same data as for preceding; 636; SMNE• 1 male; Cheorwon-gun, [7] Geunnam-myeon, Yukdan-ri; 13 Jun. 1992; D.-S. Ku leg.; 666; SMNE • 1 female; Inje-gun, [8] Buk-myeon, Yongdae-ri, Seoraksan Mountain, Baekdamsa Temple; 25 May 1993; D.-S. Ku leg.; 641; SMNE • 1 female; Hongcheon-gun, [9] Duchon-myeon, Jangnam-ri (Corn Experimantal Station); 3 Jun. 1996; June-Yeol Choi leg.; 653; SMNE • 1 male; Chuncheon-si, [10] Sinbuk-eup, Cheonjeon-ri, Cheonjeon 5-ri; 25 May 1993; D.-S. Ku leg.; 654; SMNE • 1 male; Taebaek-si, [12] Cheoram-dong; 22 Jun. 1991; D.-S. Ku leg.; 645; SMNE • 1 female; Taebaek-si, [13] Cheoram-dong, Taebaeksan Mountain; 13 May 1993; D.-S. Ku leg.; 658; SMNE. – **Gyeonggi-do** • 1 female; Gapyeong-gun, [14] Cheongpyeong-myeon, Cheongpyeong-ri, Cheongpyeong Amusement Park; 14 Jun. 1992; D.-S. Ku leg.; 652; SMNE • 2 females; Bonghwa-gun, [18] Myeongho-myeon, Gwanchang-ri; 28 May 1993; D.-S. Ku leg.; 648, 650; SMNE • 2 males; same data as for preceding; 649, 651; SMNE • 1 female; Mungyeong-si, [19] Buljeong-dong; 9 Jun. 1992; D.-S. Ku leg.; 631; SMNE. – **Chung- cheongbuk-do** • 1 female; Danyang-gun, [20] Danyang-eup, Dodam-ri; 13 May 1991; D.-S. Ku leg.; 657; SMNE. – **Chungcheongnam-do** • 1 female; Geumsan-gun, [21] Chubu-myeon, Seongdang-ri, Gaedeoksa Temple; 22 May 1993; D.-S. Ku leg.; 640; SMNE • 2 males; same data as for preceding; 642, 643; SMNE • 1 male; same data as for preceding; 644; ZISP • 1 female; Yesan-gun, [22] Deoksan-myeon, Sudeoksa Temple; 11 Aug. 1991; D.-S. Ku leg.; 630; SMNE • 1 male; Cheongyang-gun, [23] Jeongsan-myeon, Machi-ri; 15 Jun. 1992; D.-S. Ku leg.; 665; SMNE. – **Gyeongsangbuk-do** • 1 female; Gyeongsan-si, [25] Yeongnam University; 19 Apr. 1991; J.-W. Lee leg.; 663; SMNE. – **Gyeongsangnam-do** • 1 female; Changwon-si, [27] Uichang-gu, Sogye-dong, Cheonjusan Mountain; 18 Jun. 1992; D.-S. Ku leg.; 659; SMNE • 1 male; Jinju-si, [28] Gajwa-dong; 18 May 1993; D.-S. Ku leg.; 660; SMNE • 1 female; Goseong-gun, [29] Sangni-myeon, Bupo-ri; 3 May 1993; D.-S. Ku leg.; 664; SMNE. – **Jeollanam-do** • 1 female; Gurye-gun, [31] Sandong-myeon, Jwasa-ri, Jirisan Mountain (Simwon); 5 May 1993; D.-S. Ku leg.; 662; SMNE • 2 females; Yeosu-si, [32] Nam-myeon, Dumo-ri, Town Moha; 20 Jul. 1993; D.-S. Ku leg.; 646, 647; SMNE • 1 female; Yeosu-si, [34] Nam-myeon, Yeondo Island, Yeondo-ri; 21 Jul. 1993; D.-S. Ku leg.; 656; SMNE • 1 female; same data as for preceding; 655; ZISP.

**Figures 79–85.**
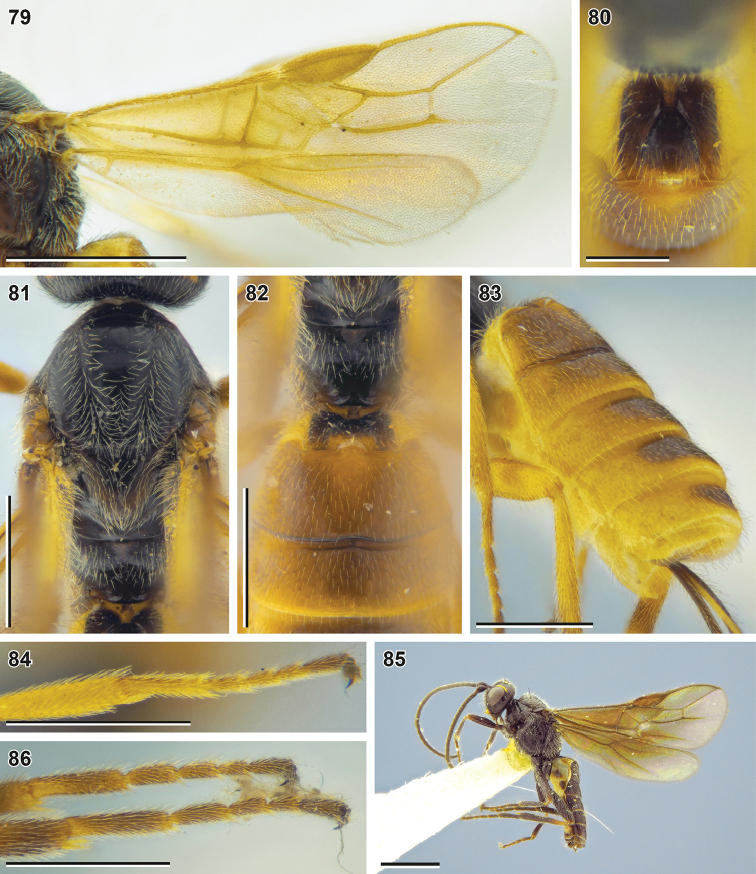
Bracon (Habrobracon) allevatus sp. nov. (**79–84** holotype, NIBR**85** male paratype, SMNE) **79** wings **80** first metasomal tergite, dorsal view **81** mesosoma, dorsal view **82** propodeum and base of metasoma, dorsal view **83** metasoma, dorsolateral view **84** fore tibia and tarsus **85** habitus, lateral view **86** hind tarsus. Scale bars: 1 mm (**79, 85**); 0.5 mm (**81–84, 86**); 0.25 mm (**80**).

##### Etymology.

The Latin adjective *allevatus* (smoothed off) refers to the strongly reduced sculpture of the body discriminating the new species from *B. variegator* Spinola.

##### Description.

**Female.** Body length 2.4–3.1 mm; fore wing length 2.4–3.1 mm.

***Head*.** Width of head (dorsal view) 1.9–2.0× its median length. Transverse diameter of eye (dorsal view) 1.6–2.0× longer than temple. Eyes with dense, short setae. OOL 2.3–2.8× Od; POL 1.4–1.9× Od; OOL 1.4–1.8× POL. Frons with deep mid-longitudinal groove. Longitudinal diameter of eye in lateral view 1.5–1.6× larger than its transverse diameter. Transverse diameter of eye (lateral view) 1.7–2.0× longer than minimum width of temple, hind margins of eye and temple broadened downwards or more or less parallel. Face width 1.6–1.8× combined height of face and clypeus; 2.4–2.9× larger than width of hypoclypeal depression. Longitudinal diameter of eye 2.2–2.5 (but 3.4× in the smallest measured female) × longer than malar space (front view); malar space 0.8–0.9× base of mandible; malar suture absent. Width of hypoclypeal depression 0.95–1.25× as large as distance from depression to eye. Clypeus not separated from face by dorsal carina, clypeal sulcus absent, dorsal clypeal margin sharp. Clypeus prominent, with protruding ventral rim, height of clypeus 0.30–0.45× width of hypoclypeal depression. Maxillary palp as long as eye height.

***Antenna*** 0.86–0.91× as long as fore wing, with 24–29 antennomeres. First flagellomere 1.6–2.0× longer than its apical width, 0.95–1.15× as long as second flagellomere. Middle and penultimate flagellomeres 1.3–1.9× and 1.6–2.0× longer than wide, respectively. Apical flagellomere spiculate.

***Mesosoma*** 1.4–1.6× longer than its maximum height. Transverse pronotal sulcus smooth, deep anteriorly and posteriorly, shallow medially. Notauli impressed, not united posteriorly, smooth. Mesoscutum widely setose on notauli and anterolaterally, widely smooth medially and latero-posteriorly. Scutellar sulcus crenulate. Mesepimeral sulcus smooth. Mesopleural pit deep, separated from mesepimeral sulcus. Median area of metanotum with incomplete median carina. Metapleural sulcus smooth or weakly crenulate. Propodeum with simple mid-longitudinal keel in apical third.

***Wings.*** Fore wing 0.95–1.10× as long as body. Pterostigma 2.4–3.6× longer than wide. Vein r arising from basal 0.4–0.5 of pterostigma length. Vein 1-R1 1.3–1.4× longer than pterostigma. Marginal cell 3.5–7.0× longer than distance from its apex to apex of wing. Vein 3-SR 1.6–2.1× longer than vein r, 0.50–0.65× as long as vein SR1, 1.1–1.4× longer than vein 2-SR. Vein 1-M 0.75–0.90× vein 1-SR+M, 1.7–2.3× vein m-cu. 2.5–3.0× longer than vein cu-a. Vein 2-SR+M 0.35–0.55× as long as vein 2-SR, 0.65–0.85× as long as vein m-cu. Vein 1-CU1 (posterior margin of discal cell) 2.6–3.2× longer than vein cu-a. Vein cu-a interstitial. Vein 2-1A of hind wing absent; vein r-m antefurcal.

***Legs.*** Fore tibia with thick setae subapically. Hind femur 3.4–4.0× longer than wide. Hind tibia ca. 1.5× longer than hind femur, without subapical row of thick setae, its inner spur 0.40–0.45× as long as hind basitarsus. Hind tarsus 0.87–0.99× as long as hind tibia. Fifth segment (without pretarsus) of hind tarsus 0.40–0.45× as long as hind basitarsus and 0.75–0.85× as long as second segment. Claws with small rectangular basal lobe.

***Metasoma*** 1.2–1.3× longer than mesosoma. Median length of first tergite (measured from petiolar tubercle) 0.90–1.15× as large as its apical width. Dorsal and dorsolateral carinae of first metasomal tergite absent. Median area of first tergite separated by smooth or weakly crenulate furrow, 0.6–0.7× apical width of tergite. Second tergite medially 1.0–1.2× as long as third tergite and 0.9–1.0× as large as apical width of first tergite, without dorsolateral impressions. Basal width of second metasomal tergite 1.3–1.8× larger than its median length. Suture between second and third tergites deep, curved and smooth or weakly crenulate. Apical margins of third to sixth tergites thick, without transverse subapical grooves. Ovipositor sheath 0.55–0.65× as long as hind tibia and 0.17–0.20× as long as fore wing. Apex of ovipositor with (sometimes weak) nodus and weak or indistinct ventral serration.

***Sculpture.*** Face granulate, frons weakly granulate, gena hardly coriaceous to smooth. Vertex, most of mesosoma and coxae smooth. Propodeum smooth, sometimes with short rugae apically. Metasoma entirely smooth or with weak granulate sculpture at most on second tergite.

***Colour.*** Body mainly brownish black, metasomal tergites sometimes brown, ventral side of metasoma pale yellow. Head with yellowish brown patches along eyes on vertex and in lower part of gena, mandible and maxillary palps yellowish brown. Apices of femora and bases of tibiae of all legs (half of hind tibia) brownish yellow. Apical margins of metasomal tergites 3–7 light-coloured. Tegulae dark brown. Wing membrane brownish darkened, lighter apically; pterostigma brown or yellowish brown, with small pale yellow patch basally, wing veins yellowish brown.

**Male.** Body length 2.0–2.4 mm; fore wing length 2.1–2.5 mm. OOL 1.1–1.3× POL. Mid-longitudinal keel developed on apical half of propodeum. Median length of first tergite (measured from petiolar tubercle) 1.2–1.3× larger than its apical width. Face sometimes smooth medially on narrow area. Maxillary palps brown or brownish yellow. Otherwise similar to female.

##### Diagnosis.

*Bracon allevatus* sp. nov. is most similar to *B. variegator* Spinola. The latter species was classified within the nominative subgenus of *Bracon* (Papp 1968, 2012) or its subgenus Habrobracon (Tobias 1986; Tobias and Belokobylskij 2000). It seems best to consider both species in *Habrobracon* because they share a number of characteristic character states (the malar suture is absent; basal lobes of tarsal claws not protruding or acutely protruding (not lamelliform); in the fore wing, the vein 1-SR+M is straight, the vein 3-SR usually is no longer than vein 2-SR (0.6–1.2×), the vein 2-SR+M is long, 0.6–1.2× as long as vein 3-SR; the dorsal carinae of the first metasomal tergite are absent, the lateral carinae are absent or very weakly defined; the ovipositor sheath is at most somewhat longer than the hind tibia, shorter than half of the fore wing length; the granulate sculpture tends to be more or less developed on body). In addition, *Habrobracon* was considered either a separate genus (Quicke 1987; Papp 2012; Kittel and Maeto 2019) or a subgenus of *Bracon* (Tobias 1986; van Achterberg and Polaszek 1996; Tobias and Belokobylskij 2000). Here the latter hypothesis is accepted because a number of very similar species are known in the subgenera *Sculptobracon* (*B. yakui* Watanabe, 1937 and *B. obsoletus* Li, He & Chen, 2016) and *Bracon* s. str. (*B. concavus* species group). Until the differences between the latter taxa and *Habrobracon* are sorted out, we prefer to keep *Habrobracon* as a subgenus of *Bracon*. *B. allevatus* sp. nov. maybe also compared with *B. kasparyani* distributed in the same region. The differences between three species are listed in the key below.

**Table:** 

1	Malar suture weakly impressed (Fig. 87). Face (almost) smooth. Notauli deep or impressed anteriorly, smoothened posteriorly (Fig. 88). Claws with acute angularly protruding basal lobe. Vein 2-SR+M 0.2–0.3× as long as vein 2-SR (Fig. 97). Body almost entirely smooth. Dorsolateral carinae of first metasomal tergite distinctly separated (Fig. 92)	**Bracon (Bracon) kasparyani Samartsev, 2018**
–	Malar suture absent (Figs 73, 89). Face distinctly granulate. Notauli absent or shallowly impressed (Figs 81, 90). Claws with rectangular, not protruding basal lobe (Fig. 84). Vein 2-SR+M 0.35–0.80× as long as vein 2-SR (Figs 79, 98)..	**2**
2	Metasoma entirely, vertex and frons distinctly granulate (Figs 90, 93). Propodeum granulate, without mid-longitudinal keel (Fig. 93). Marginal cell 2.0–3.5× longer than distance from its apex to apex of wing (Fig. 98). Propodeal spiracle located behind middle of propodeum (lateral view). Notauli not impressed (Fig. 90)	**B. (Habrobracon) variegator Spinola, 1808**
–	Metasoma mainly smooth, with weak granulate sculpture only on second tergite (Fig. 80, 82, 83); vertex smooth, frons weakly granulate (Fig. 71). Propodeum smooth, usually with simple mid-longitudinal keel in its apical half (Figs 81, 82). Marginal cell 3.5–7.0× longer than distance from its apex to apex of wing (Fig. 79). Propodeal spiracle located in middle of propodeum (lateral view; Fig. 72). Notauli shallowly impressed (Fig. 81)	**B. (H.) allevatus sp. nov.**

**Figures 87–98.**
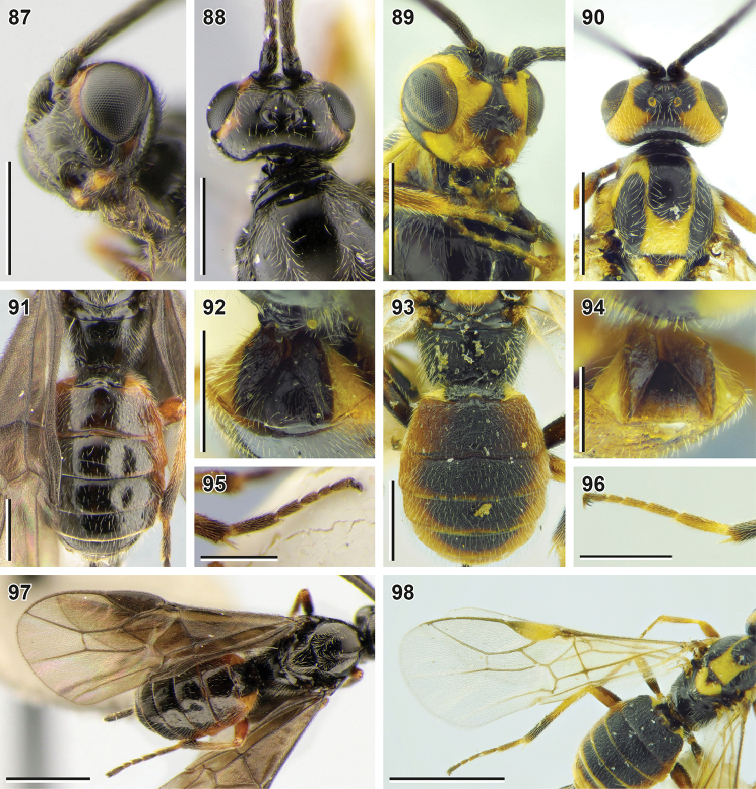
*Bracon kasparyani* Samartsev, 2018 (**87, 91, 97** holotype, ZISP**88, 92, 95** female paratype, ZISP), *Bracon variegator* Spinola, 1808 (**89, 90, 93, 94, 96, 98** female, ZISP) **87, 89** head, ventrolateral view **88, 90** head and mesoscutum, dorsal view **91, 93** metasoma, dorsal view **92, 94** first metasomal tergite, dorsal view **95, 96** hind tarsus **97, 98** fore wing. Scale bars: 1 mm (**97, 98**); 0.5 mm (**87–93, 95, 96**); 0.25 mm (**87–93, 95, 96**).

#### Bracon (Osculobracon) perspicillatus sp. nov.

Taxon classificationAnimaliaHymenopteraBraconidae

8E4E4EE8-626B-5563-98D3-649BB2E2AA9A

http://zoobank.org/5AFDBB69-7E49-460D-9A33-A7841DB329D5

Figs 99–106 , 107–114

##### Type material.

***Holotype.*** South Korea – **Gangwon-do** • female; Goseong-gun, [5] Ganseong-eup, Jinbu-ri; 12 Jun. 1992; D.-S. Ku leg.; 306; NIBR.

***Paratypes.*** 2 females, 4 males. South Korea – **Gangwon-do** • 1 male; same data as for holotype; 307; ZISP • 1 male; same data as for holotype; 308; SMNE • 1 female; Goseong-gun, [4] Geojin-eup, Naengcheon-ri, Geonbongsa Temple; 25 May 1993; D.-S. Ku leg.; 278; SMNE • 2 males; same data as for preceding; 313, 314; SMNE • 1 female; same data as for preceding; 304; ZISP.

**Figures 99–106.**
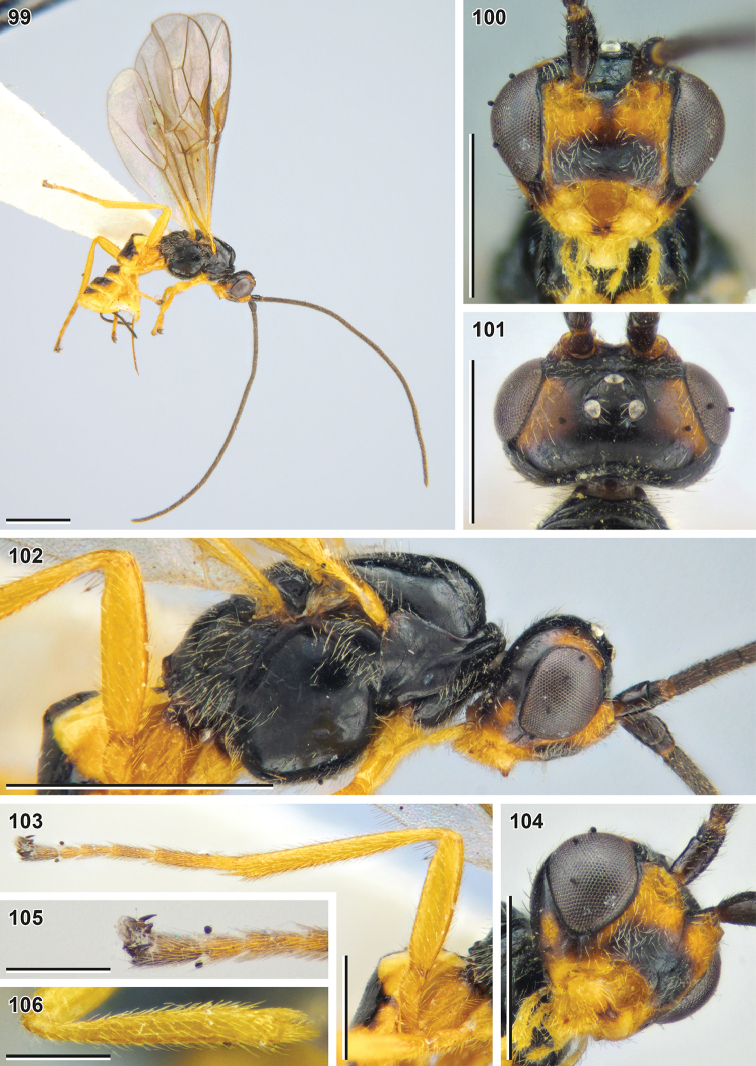
Bracon (Osculobracon) perspicillatus sp. nov. (holotype, NIBR) **99** habitus, lateral view **100** head, front view **101** head, dorsal view **102** head and mesosoma, lateral view **103** hind leg **104** head, ventrolateral view **105** apex of hind tarsus **106** fore tibia. Scale bars: 1 mm (**99, 102**); 0.5 mm (**100, 101, 103, 104**); 0.25 mm (**105, 106**).

##### Etymology.

The name *perspicillatus* (from Latin *perspicillum* for spectacles) refers to a pair of light patches on the face below toruli which characterise the species.

**Figures 107–114.**
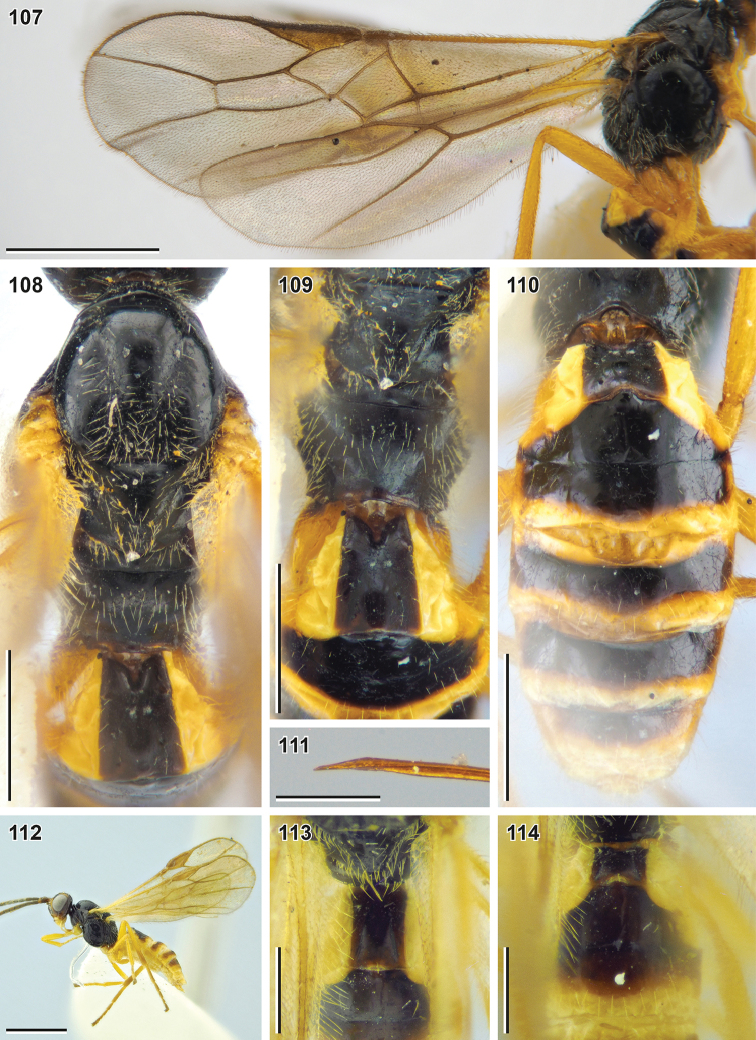
Bracon (Osculobracon) perspicillatus sp. nov. (**107–111** holotype, NIBR, **112–114** male paratype, SMNE) **107** wings **108** mesosoma, dorsal view **109** propodeum and first metasomal tergite, dorsal view **110** metasoma, dorsal view **111** apex of ovipositor **112** habitus, lateral view **113** first metasomal tergite, dorsal view **114** second and third metasomal tergites, dorsal view. Scale bars: 1 mm (**107, 112**); 0.5 mm (**108–110**); 0.25 mm (**111, 113, 114**).

##### Description.

**Female.** Body length 2.3–3.4 mm; fore wing length 2.6–3.7 mm.

***Head*.** Width of head (dorsal view) 1.8–1.9× its median length. Transverse diameter of eye (dorsal view) 1.7–1.8× longer than temple. Eyes with sparse, short setae. OOL 2.4–3.0× Od; POL 1.3–1.9× Od; OOL 1.5–1.8× POL. Frons with deep mid-longitudinal groove. Longitudinal diameter of eye in lateral view 1.4–1.5× larger than its transverse diameter. Transverse diameter of eye (lateral view) 1.8–2.4× longer than minimum width of temple, hind margins of eye and temple broadened downwards. Face width 1.4–1.5× combined height of face and clypeus; 2.3–2.6× larger than width of hypoclypeal depression. Longitudinal diameter of eye 2.4–2.8× longer than malar space (front view); malar space 0.87–0.92× base of mandible. Malar suture deep and smooth. Width of hypoclypeal depression 1.0–1.3× larger than distance from depression to eye. Clypeus not separated from face by dorsal carina, flattened, with not protruding ventral rim, height of clypeus 0.30–0.35× width of hypoclypeal depression; clypeal sulcus smoothened. Maxillary palp longer than eye, but shorter than head.

***Antenna*** ca. 1.2× longer than fore wing, with 32–40 antennomeres. First flagellomere 2.0–2.2× longer than its apical width, 1.0–1.1× longer than second flagellomere. Middle and penultimate flagellomeres 1.6–2.0× and 1.8–2.2× longer than wide, respectively. Apical flagellomere spiculate.

***Mesosoma*** ca. 1.6× longer than its maximum height. Transverse pronotal sulcus smoothened. Notauli smooth, impressed anteriorly, smoothened and not united posteriorly. Mesoscutum setose only on notauli. Scutellar, mesepimeral and metapleural sulci smooth, mesopleural pit indistinct. Median area of metanotum with incomplete median carina. Mid-longitudinal keel on propodeum absent.

***Wings.*** Fore wing 1.0–1.1× longer than body. Pterostigma 3.2–3.7× longer than wide. Vein r arising from basal 0.40–0.45 of pterostigma length. Vein 1-R1 1.3–1.6× longer than pterostigma. Marginal cell 7.5–9.7× longer than distance from its apex to apex of wing. Vein 3-SR 2.3–2.7× longer than vein r, 0.60–0.65× as long as vein SR1, 1.5–1.7× longer than vein 2-SR. Vein 1-M 0.75–0.85× vein 1-SR+M, 1.5–1.7× vein m-cu. 2.1–2.2× longer than vein cu-a. Vein 2-SR+M 0.16–0.22× as long as vein 2-SR, 0.23–0.38× as long as vein m-cu. Vein 1-CU1 (posterior margin of discal cell) 2.3–2.8× longer than vein cu-a. Vein cu-a interstitial. Vein 2-1A of hind wing absent or very short; vein r-m strongly antefurcal.

***Legs.*** Fore tibia with sparse longitudinal and dense transverse apical rows of thick setae. Hind femur 3.8–3.9× longer than wide. Hind tibia 1.5–1.7× longer than hind femur, without subapical row of thick setae, its inner spur 0.23–0.30× as long as hind basitarsus. Hind tarsus 0.95–1.00× as long as hind tibia. Fifth segment (without pretarsus) of hind tarsus 0.45–0.50× as long as hind basitarsus and 0.80–0.85× as long as second segment. Claws with large, protruding and blunt basal lobes.

***Metasoma*** 1.2–1.4× longer than mesosoma. Median length of first tergite (measured from petiolar tubercle) 1.3–1.5× larger than its apical width. Dorsolateral and dorsal carinae of first metasomal tergite absent. Median area of first tergite separated by smooth furrow, 0.6–0.7× apical width of tergite. Second tergite sclerotised in anterior 0.85–0.95, medially 0.9–1.0× as long as third tergite and 0.85–1.05× as large as apical width of first tergite. Basal width of second metasomal tergite 1.8–1.9× larger than its median length. Anterolateral margin of second metasomal tergite shortly de-sclerotised. Suture between second and third tergites thin, shallow, weakly curved and smooth. Apical margins of third to sixth tergites largely de-sclerotised. Ovipositor sheath 0.50–0.75× as long as hind tibia and 0.16–0.21× as long as fore wing. Apex of ovipositor with weak nodus and weak or absent ventral serration.

***Sculpture.*** Body completely smooth.

***Colour.*** Body brownish black or brown. Head with more or less developed brownish yellow patches near eyes (on face, vertex and in lower part of gena), below toruli and on oral parts. Maxillary palps yellow. Tegulae, legs and de-sclerotised parts of metasoma yellow to brownish yellow or yellowish brown. Wing membrane weakly darkened, basally yellowish; pterostigma and wing veins brown or yellowish brown.

**Male.** Body length 2.1–2.6 mm; fore wing length 2.5–2.7 mm. Width of head (dorsal view) 1.6–1.8× its median length. Transverse diameter of eye (dorsal view) 1.8–2.2× longer than temple. Hind margins of eye and temple less broadened downwards (subparallel). Mesosoma 1.5–1.8× longer than its maximum height. Fore wing vein 3-SR 2.7–2.9× longer than vein r, 0.61–0.74× as long as vein SR1, 1.6–1.9× longer than vein 2-SR. Second tergite sclerotised in anterior 0.75–0.90, its basal width 1.2–1.8× larger than median length. Otherwise similar to female.

##### Diagnosis.

The new species is most similar to *Bracon cingulator* Szépligeti, *B. koreanus* Papp, and *B. osculator* Nees, which also have the entirely smooth body and not shortened marginal cell of the fore wing. The differences between these species are listed in the key below (the characters for *B. cingulator* and *B. osculator* are given on the basis of an unpublished dataset).

**Table:** 

1	Median length of first metasomal tergite (measured from spiracle) 0.6–0.9× as large as its apical width (being measured from petiolar tubercle 0.85–1.20× as large as its apical width; Fig. 121). Malar space 0.90–1.05× base of mandible (Fig. 115). Basal width of second metasomal tergite 1.0–1.8× larger than its median length (Fig. 124). Head and most of mesosoma reddish yellow. Pterostigma yellow with brown patch apically. Longitudinal diameter of eye 2.3–2.4× longer than malar space (front view). Second metasomal tergite medially 0.60–0.75× as long as third tergite. Longitudinal diameter of eye 0.85–0.90× as large as face width (front view). Antenna ca. 1.1× longer than fore wing	**Bracon (Osculobracon) koreanus Papp, 1998**
–	Median length of first metasomal tergite (measured from spiracle) 0.95–1.15× as large as its apical width (being measured from petiolar tubercle 1.2–1.6× larger than its apical width; Figs 108, 122, 123). Malar space 0.70–0.95× base of mandible (Figs 100, 116, 117). Basal width of second metasomal tergite 1.8–2.8× larger than its median length (Figs 110, 125, 126). Head and most of mesosoma usually black. Pterostigma entirely brown or yellow	**2**
2	Second metasomal tergite medially 0.9–1.0× as long as third tergite (Fig. 110). Antenna with 32–40 antennomeres, ca. 1.2× longer than fore wing (Fig. 99). Mesosoma ca. 1.6× longer than its maximum height (Fig. 102). Face with light-coloured patches below toruli (Fig. 100). Sclerotised part of third metasomal tergite 0.87–0.95× as long as second metasomal tergite medially. Vein 3-SR 1.5–1.7× longer than vein 2-SR. Longitudinal diameter of eye 2.4–2.8× longer than malar space (front view)	**B. (O.) perspicillatus sp. nov.**
–	Second metasomal tergite medially 0.70–0.75× as long as third tergite (Figs 125, 126). Antenna with 25–28 antennomeres, 0.97–1.13× as long as fore wing. Mesosoma 1.4–1.5× longer than its maximum height. Face without light-coloured patches below toruli (Figs 116, 117)	**3**
3	Median length of first metasomal tergite (measured from spiracle) usually 1.0–1.2× as large as its apical width (Fig. 122). Width of hypoclypeal depression usually 1.15–1.30× as large as distance from depression to eye (Fig. 116). Longitudinal diameter of eye usually 2.9–3.4× longer than malar space (front view). Basal width of second metasomal tergite usually 1.9–2.4× larger than its median length (Fig. 125)	**B. (O.) cingulator Szépligeti, 1901**
–	Median length of first metasomal tergite (measured from spiracle) usually 0.9–1.0× as large as its apical width (Fig. 123). Width of hypoclypeal depression usually 1.00–1.15× larger than distance from depression to eye (Fig. 117). Longitudinal diameter of eye usually 2.4–2.8× longer than malar space (front view). Basal width of second metasomal tergite usually 2.3–2.8× larger than its median length (Fig. 126)	**B. (O.) osculator Nees, 1811**

**Figures 115–126.**
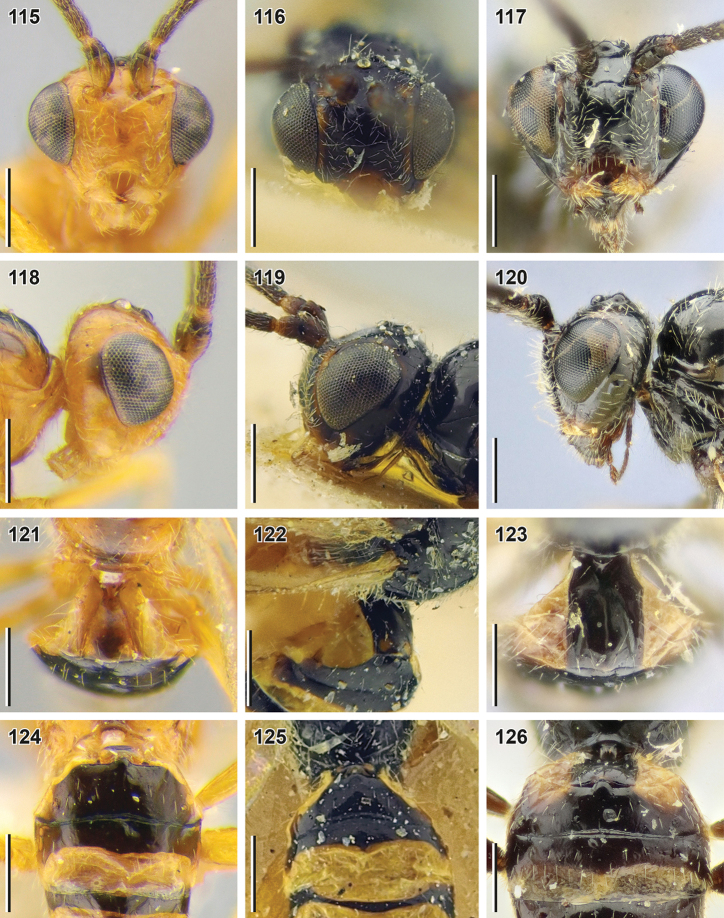
Bracon (Osculobracon) koreanus Papp, 1998 (**115, 118, 121, 124** holotype, HNHM), B. (O.) cingulator Szépligeti, 1901 (**116, 119, 122, 125** holotype, HNHM) and B. (O.) osculator Nees, 1811 (**117, 120, 123, 126** lectotype of *B. coniferarum* Fahringer, 1928, MNB) **115–117** head, front view **118–120** head, lateral view **121–123** first metasomal tergite **124–126** second and third metasomal tergites, dorsal view. Scale bars: 0.25 mm.

#### Genus Syntomernus

Taxon classificationAnimaliaHymenopteraBraconidae

Enderlein, 1920

707D66B5-0FC3-5B9B-A6FE-1C169DED838B

Syntomernus Enderlein 1920: 121 (type species: Syntomernus pusillus Enderlein, 1920). Shenefelt 1978: 1728; Quicke 1987: 89 (in key); 132; van Achterberg et al. 2009: 664.
Ficobracon van Achterberg & Weiblen, 2000: 52 (type species: Ficobracon brusi van Achterberg & Weiblen, 2000). Wei et al. 2013: 466; syn. nov. Syntomernus brusi (van Achterberg & Weiblen, 2000), comb. nov.

##### Remarks.

The members of the *Bracon asphondyliae* species group (Maetô 1991) fit well the diagnosis of *Ficobracon*, while the latter genus must be synonymised with the genus *Syntomernus*. Most characters indicated as diagnostic for two latter genera (van Achterberg and Weiblen 2000) show an overlap. The only exception is the difference in the setosity of mesoscutum (the median lobe of mesoscutum medially setose in *Syntomernus* and glabrous in *Ficobracon*), but this character is not considered strong enough to warrant generic status of *Ficobracon*. In addition, members of the species attributed here to the genus *Syntomernus* parasitise ecologically similar hosts. Braconid wasps of the *asphondyliae* species group attack cecidomiid gall midges (Maetô 1991; Matsuo et al. 2016), *Syntomernus shoreatus* van Achterberg & Ng, 2009 uses larvae inside dipterocarp fruits (van Achterberg et al. 2009), the members of *Ficobracon* have been reared from fig syconia (van Achterberg and Weiblen 2000; Wei et al. 2013) and *Syntomernus kashmirensis* (Maqbool, Akbar & Wachkoo, 2018), comb. nov. is known to be phytophagous on the syconium tissues (Maqbool et al. 2018). The main character separating *Syntomernus* from *Bracon* is the presence of anterolateral areas on third metasomal tergite. The full diagnosis of the genus is presented below.

##### Diagnosis.

***Head*** transverse, its width (dorsal view) 1.7–2.1× its median length, with transverse diameter of eye 1.7–3.0× longer than temple. Clypeus without or with weak dorsal carina, clypeal sulcus absent, dorsal clypeal margin sharp or smoothened. Vertex without mid-longitudinal sulcus. Malar suture absent or weakly impressed. Hind margins of eye and temple (in lateral view) more or less broadened downwards.

***Antenna*.** Dorsal side of scape (lateral view) longer than its ventral side. Antennae with elongate segments, first flagellomere 2–4× longer than its apical width, middle and penultimate flagellomeres 1.7–2.5× longer than wide.

***Mesosoma*** 1.1–1.5× longer than its maximum height. Median lobe of mesoscutum evenly setose or setose only on notauli and posteriorly. Notauli usually deep anteriorly, smoothened or absent and not united posteriorly. Precoxal sulcus absent or vaguely impressed. Mesopleural pit weak or almost indistinct. Mesepimeral sulcus smooth or weakly crenulate, metapleural sulcus smooth. Propodeum with simple and high mid-longitudinal keel developed at least in its apical half and with mid-longitudinal impression in its basal half.

***Legs.*** Hind tibia without subapical row of thick setae (at most with two thick setae subapically). Claws with moderately large, not protruding (rounded) or angularly protruding (acute or blunt) basal lobe.

***Wings.*** Angle between veins C+SC+R and 1-SR ca. 50–70 degrees. Marginal cell of fore wing not shortened, 7–24× longer than distance from its apex to apex of wing. Vein SR1 distinctly elongate. Vein 3-SR 0.22–0.42× as long as vein SR1, 0.75–1.50× as long as vein 2-SR. Vein 1-SR+M more or less curved anteriorly. Hind wing with basally evenly setose membrane. Vein 2-1A of hind wing absent or very short.

***Metasoma*** with six coarsely sculptured tergites. First metasomal tergite with distinct, often deep crenulate mid-longitudinal impression and more or less developed dorsal and dorsolateral carinae. Second metasomal tergite without anterolateral, posteriorly diverging grooves; with dorsolateral impressions more or less deep, crenulated, usually with strong posteriorly converging carinae along their proximal margin. Median area of second metasomal tergite elongate-triangle or longitudinal, with sharp margin. Spiracle of second metasomal tergite located in middle or behind middle of tergite. Suture between second and third tergites deep and curved. Anterolateral areas of third tergite always developed, large and separated by crenulate suture. Apical margins of third to sixth tergites thick, with deep crenulate transverse subapical grooves. Ovipositor sheath 1.4–3.6× longer than hind tibia, 0.4–1.0× as long as fore wing. Apex of ovipositor with developed nodus and ventral serration.

A key to the species of the genus *Syntomernus* from Eastern Palaearctic is presented below. *Syntomernus codonatus* and *S. rhiknosus* from the Oriental part of China were also included there while five species described in Chen and Yang (2006) could not be included because the types were not available, and the descriptions are insufficient for inclusion.

**Table:** 

1	Ovipositor sheath ca. 0.4× as long as fore wing (Fig. 157). Longitudinal diameter of eye 2.0× longer than malar space (front view; Fig. 158). Vertex weakly granulate. Median lobe of mesoscutum anteriorly evenly setose (Fig. 159)	***Syntomernus pusillus* Enderlein, 1920**
–	Ovipositor sheath 0.60–0.95× as long as fore wing (Figs 127, 142, 165, 176). Longitudinal diameter of eye 2.2–3.8× longer than malar space (front view; Figs 128, 143, 164, 169, 175). Vertex smooth. Median lobe of mesoscutum anteriorly glabrous	**2**
2	Ovipositor sheath 2.2× longer than hind tibia, 0.6× as long as fore wing (Fig. 142). Third–sixth metasomal tergites rugose (Figs 155, 156). Suture between second and third tergites strongly curved medially. Fifth segment (without pretarsus) of hind tarsus 1.2× longer than second segment (Fig. 148)	***S. scabrosus* sp**. **nov.**
–	Ovipositor sheath 2.7–3.6× longer than hind tibia, 0.67–0.95× as long as fore wing. Third–sixth metasomal tergites with weak papillary-like sculpture or almost smooth (only third tergite longitudinally rugose in some *S. asphondyliae* ; Figs 136, 173, 178). Suture between second and third tergites weakly curved medially	**3**
3	Second metasomal tergite medially 0.85–1.05× as long as third tergite (Fig. 166). Basal width of second metasomal tergite 2.2–2.5× larger than its median length. Third and following metasomal tergites (almost) smooth. Frons and vertex black (Fig. 163). – Dorsolateral impressions of second tergite shallow	***S. sunosei* (Maeto, 1991), comb. nov. (*B. flaccus* Papp, 1996, syn. nov.)**
–	Second metasomal tergite medially 1.1–1.3× longer than third tergite (Figs 136, 173, 178). Basal width of second metasomal tergite 1.3–2.0× larger than its median length. Third and following metasomal tergites distinctly sculptured, with weak papillary-like sculpture. Frons and vertex light-coloured (Figs 131, 170, 174)	**4**
4	Fifth segment (without pretarsus) of hind tarsus 0.75–0.95× as long as second segment (Fig. 135)	**5**
–	Fifth segment (without pretarsus) of hind tarsus 1.0–1.2× longer than second segment (Fig. 172)	**6**
5	Antenna with 34–36 antennomeres. Face width 2.1–2.2× larger than width of hypoclypeal depression (Fig. 128). Pterostigma brown with large yellow patch basally (Fig. 134). Anterolateral areas of second metasomal tergite smooth (Fig. 136). Body entirely yellow. – Antenna 1.0–1.1× longer than fore wing	***S. flavus* sp. nov.**
–	Antenna with 20–23 antennomeres. Face width ca. 2.5× larger than width of hypoclypeal depression. Pterostigma brown (fig. 7 in Wei et al. 2013). Anterolateral areas of second metasomal tergite rugulose (ibid, fig. 6). Body with developed dark pattern, hind tibia apically and first metasomal tergite black	***S. codonatus* (Huang & van Achterberg, 2013), comb. nov.**
6	Median area of second metasomal tergite narrower, parallel-sided and weakly elevated (Figs 171, 173); dorsolateral impressions of second tergite weak. Longitudinal diameter of eye 3.4–3.8× longer than malar space (front view; Fig. 169). Malar suture absent. Ovipositor sheath ca. 3× longer than hind tibia	***S. tamabae* (Maeto, 1991), comb. nov.**
–	Median area of second metasomal tergite wider, elongate-triangle and strongly elevated (Figs 177, 178; fig. 25 in Wei et al. 2013); dorsolateral impressions deep. Longitudinal diameter of eye 2.4–3.0× longer than malar space (front view; Fig. 175). Malar suture usually weakly impressed. Ovipositor sheath 3.3–3.6× longer than hind tibia	**7**
7	Vein 3-SR 0.94–1.17× as long as vein 2-SR (Fig. 176). Vein 2-SR 1.8–2.0× longer than vein r	***S. asphondyliae* (Watanabe, 1940), comb. nov.**
–	Vein 3-SR ca. 1.5× longer than vein 2-SR (fig. 22 in Wei et al. 2013). Vein 2-SR ca. 1.1× longer than vein r	***S. rhiknosus* (Huang & van Achterberg, 2013), comb. nov.**

#### Syntomernus flavus sp. nov.

Taxon classificationAnimaliaHymenopteraBraconidae

6A1CD802-30CD-5001-A8ED-2D41C79341FD

http://zoobank.org/D0039386-8036-49EB-BDE3-F80D39FF1FA9

Figs 127–133 , 134–141

##### Type material.

***Holotype.*** South Korea – **Gyeonggi-do** • female; Gapyeong-gun, [14] Cheongpyeong-myeon, Cheongpyeong-ri, Cheongpyeong Amusement Park; 14 Jun. 1992; D.-S. Ku leg.; 541; NIBR.

***Paratypes*.** 3 females, 1 male. South Korea – **Gangwon-do** • 1 female; Yeongwol-gun, [11] Kimsatgat-myeon, Nae-ri, Town Gijeon; 28 May 1998; Jeong-Gyu Kim leg.; 20; SMNE. – **Gyeonggi-do** • 1 female; Suwon-si, [15] Gwonseon-gu, Seodun-dong, Yeogisan Mountain; 14 Aug. 1995; J.Y. Choi leg.; Malaise trap; 1201; SMNE. – **Gyeongsangnam-do** • 1 female; Geochang-gun, [26] Geochang-eup, Songjeong-ri; 35.6712, 127.885; 3 Jun. 2019; K. Samartsev leg.; forest on a mountain; B0080; ZISP • 1 male; Geoje-si, [30] Dongbu-myeon, Hakdong-ri; 23 Jun. 1990; D.-S. Ku leg.; 18; SMNE.

**Figures 127–133.**
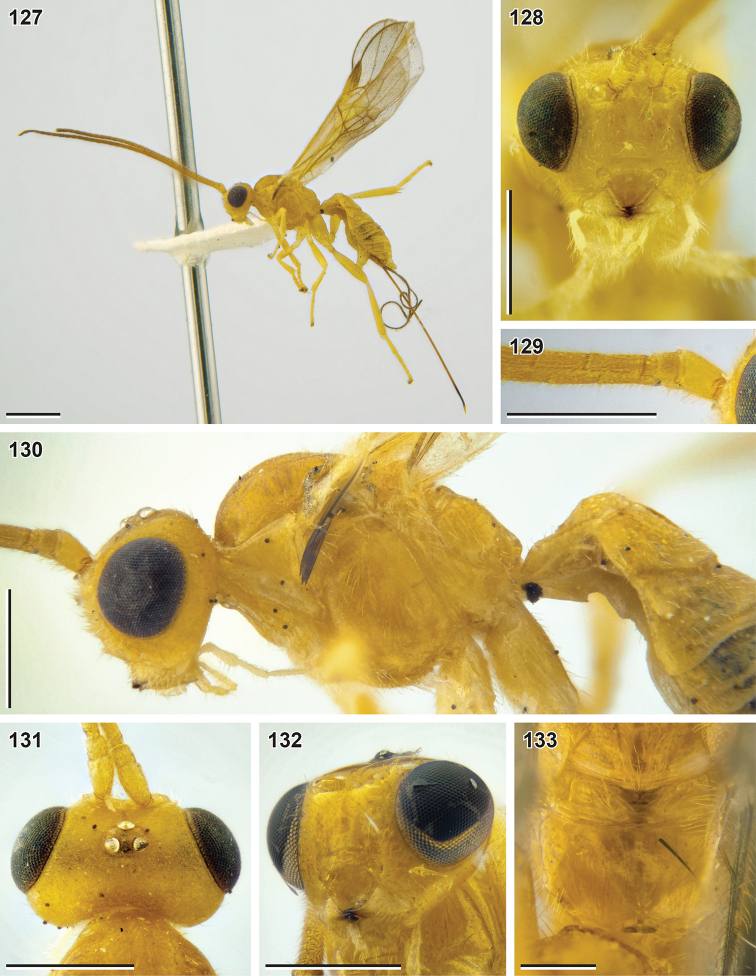
*Syntomernus flavus* sp. nov. (**127–131** holotype, NIBR**132, 133** paratype, female, SMNE) **127** habitus, lateral view **128** head, front view **129** base of antenna **130** head and mesosoma, lateral view **131** head, dorsal view **132** head, ventrolateral view **133** propodeum, dorsal view. Scale bars: 1 mm (**127**); 0.5 mm (**128–132**); 0.25 mm (**133**).

##### Etymology.

The Latin *flavus* for (pale) yellow refers to entirely light-coloured body characterising the new species.

**Figures 134–141.**
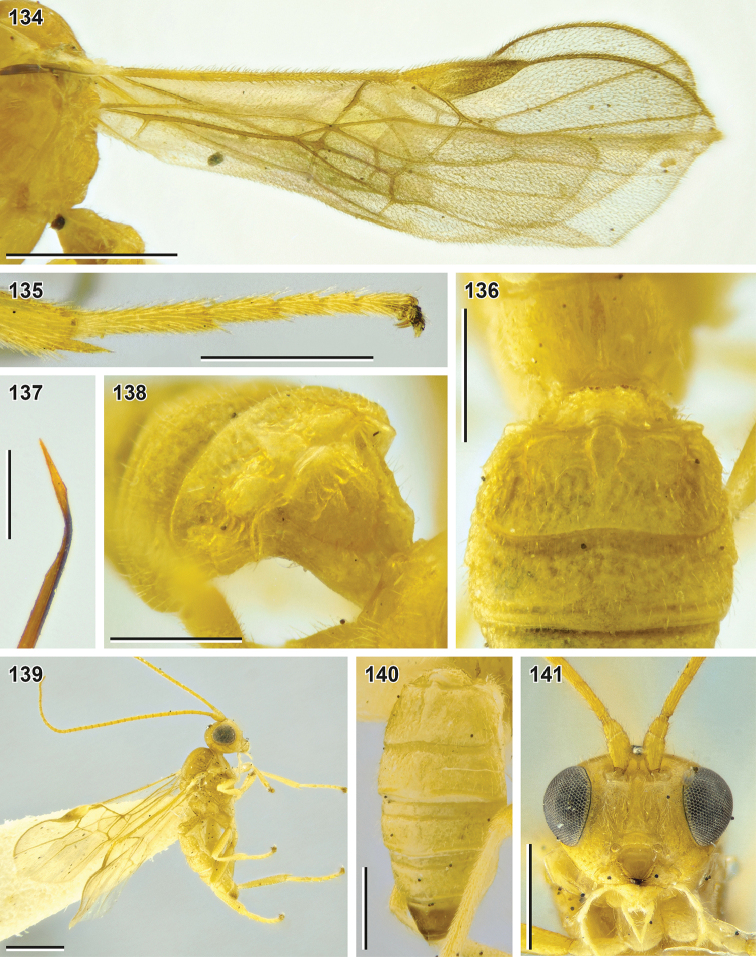
*Syntomernus flavus* sp. nov. (**134–136** holotype, NIBR**139–141** male paratype, SMNE) **134** wings **135** hind tarsus **136** second and third metasomal tergites, dorsal view **137** apex of ovipositor **138** first and second metasomal tergites, dorsolateral view **139** habitus, lateral view **140** metasoma, dorsolateral view **141** head, front view. Scale bars: 1 mm (**134, 139**); 0.5 mm (**135–138, 140, 141**); 0.25 mm (**137**).

##### Description.

**Female.** Body length 3.3–3.8 mm; fore wing length 3.9–4.0 mm.

***Head*.** Width of head (dorsal view) 2.0–2.1× its median length. Transverse diameter of eye (dorsal view) 2.2–2.8× longer than temple. Eyes with sparse, short setae. OOL 2.7–3.1× Od; POL 1.0–1.1× Od; OOL 2.5–2.8× POL. Frons with deep mid-longitudinal groove. Longitudinal diameter of eye in lateral view 1.2–1.3× larger than its transverse diameter. Transverse diameter of eye (lateral view) 2.2–2.9× longer than minimum width of temple, hind margins of eye and temple parallel to broadened downwards. Face width 1.3–1.5× combined height of face and clypeus; 2.1–2.2× larger than width of hypoclypeal depression. Longitudinal diameter of eye 2.5–2.7× longer than malar space (front view); malar space 0.85–0.92× base of mandible. Malar suture absent. Width of hypoclypeal depression 1.1–1.2× larger than distance from depression to eye. Clypeus separated from face by weak dorsal carina, flattened, with protruding ventral rim, height of clypeus 0.3–0.4× width of hypoclypeal depression, clypeal sulcus absent, dorsal clypeal margin sharp. Maxillary palp longer than eye, but shorter than head.

***Antenna*** 1.0–1.1× longer than fore wing, with 34–36 antennomeres. First flagellomere 3.0–3.2× longer than its apical width, 1.2–1.3× longer than second flagellomere. Middle and penultimate flagellomeres 1.8–1.9× and 1.9–2.1× longer than wide, respectively. Apical flagellomere spiculate.

***Mesosoma*** 1.4× longer than its maximum height. Transverse pronotal sulcus smooth, deep anteriorly and posteriorly, smoothened medially. Notauli deep anteriorly, smoothened or absent posteriorly, not united. Mesoscutum setose on notauli and medio-posteriorly, anteromedially widely glabrous. Scutellar sulcus crenulate. Mesepimeral sulcus smooth or weakly crenulate, mesopleural pit weak, furrow-like. Median area of metanotum (dorsal view) with incomplete median carina. Metapleural sulcus smooth. Mid-longitudinal keel developed in apical half of propodeum, simple and high. Propodeal spiracle round, located behind middle of propodeum.

***Wings.*** Fore wing 1.1–1.2× longer than body. Pterostigma 3.1–3.7× longer than wide. Vein r arising from basal 0.35–0.40 of pterostigma. Vein 1-R1 1.4–1.6× longer than pterostigma. Marginal cell 10–25× longer than distance from its apex to apex of wing. Vein 3-SR 1.4–2.1× longer than vein r, 0.24–0.27× as long as vein SR1, 0.8–1.1× as long as vein 2-SR. Vein 1-M 0.65–0.70× vein 1-SR+M, 1.9–2.5× vein m-cu. 1.9–2.4× longer than vein cu-a. Vein 2-SR+M 0.10–0.25× as long as vein 2-SR, 0.25–0.50× as long as vein m-cu. Vein 1-CU1 (posterior margin of discal cell) 2.8–3.6× longer than vein cu-a. Vein cu-a interstitial or weakly postfurcal. Vein 2-1A of hind wing absent or very short; vein r-m weakly antefurcal.

***Legs.*** Fore tibia with weakly thickened longitudinal and transverse apical rows of long setae. Hind femur 3.8–4.2× longer than wide. Hind tibia 1.3× longer than hind femur, without subapical row of thick setae, its inner spur 0.4–0.5× as long as hind basitarsus. Hind tarsus 0.90–0.95× as long as hind tibia. Fifth segment (without pretarsus) of hind tarsus 0.40–0.45× as long as hind basitarsus and 0.87–0.94× as long as second segment. Claws with large, protruding and blunt basal lobes.

***Metasoma*** 1.2–1.4× longer than mesosoma. Median length of first tergite (measured from petiolar tubercle) 0.75–0.90× as large as its apical width. Dorsolateral carinae of first metasomal tergite developed, dorsal carinae complete. Median area of first tergite separated by rugose furrow, 0.6–0.7× apical width of tergite, with distinct mid-longitudinal impression. Second tergite medially 1.1–1.2× longer than third tergite and 0.65–0.85× as large as apical width of first tergite. Basal width of second metasomal tergite 1.7–2.0× larger than its median length. Median area of second tergite strongly elevated, elongate triangular, with sharp crenulate margin. Anterolateral areas of second tergite wide, transverse, rounded, weakly elevated, with crenulated margin. Dorsolateral impressions of second tergite deep, s-shaped, crenulated. Spiracle of second metasomal tergite located in middle of tergite. Suture between second and third tergites deep and wide, curved and rugose. Apical margins of third to sixth tergites thick, with deep, weakly crenulate transverse subapical grooves. Ovipositor sheath 2.7–3.1× longer than hind tibia and 0.79–0.86× as long as fore wing. Apex of ovipositor with developed nodus and ventral serration.

***Sculpture.*** Most of head and mesosoma smooth. Face weakly granulate; gena smooth or weakly granulate in lower part, malar space granulate, frons smooth or weakly granulate. First metasomal tergite laterally smooth, its median area posteriorly rugose. Second tergite medially areolate-rugose, with smooth hind margin and elevated areas. Third–sixth tergites with weak papillary-like sculpture.

***Colour.*** Body brownish yellow. Scape yellow, flagellum yellowish brown, apically darkening. Maxillary palps, fore coxa and tegulae pale yellow or yellow. Wing membrane weakly darkened, darker apically; pterostigma brown with large yellow patch basally, wing veins yellowish brown.

**Male.** Body length 3.2 mm; fore wing length 3.3 mm. Longitudinal diameter of eye 2.2× longer than malar space (front view); malar space 0.8× base of mandible. Antenna 1.3× longer than fore wing, with 35 antennomeres. First flagellomere 4.1× longer than its apical width. Middle flagellomeres 2.5× longer than wide. Pterostigma 2.4× longer than wide. Median length of first tergite (measured from petiolar tubercle) 0.95× as large as its apical width.

##### Diagnosis.

The new species is remarkable by the light colouration of body, basally yellow and apically brown pterostigma, weakly sculptured elevated areas of second metasomal tergite and glabrous median lobe of mesoscutum.

#### Syntomernus scabrosus sp. nov.

Taxon classificationAnimaliaHymenopteraBraconidae

E106F930-124C-52F4-9EEB-10E7F3B5C035

http://zoobank.org/3ADBA024-0B63-4636-A909-C99333A5E75B

Figs 142–152 , 153–156

##### Type material.

***Holotype.*** South Korea – **Gangwon-do** • 1 female; Yeongwol-gun, [12] Kimsatgat-myeon, Nae-ri, Town Gijeon; 28 May 1998; Jeong-Gyu Kim leg.; 540; NIBR.

**Figures 142–152.**
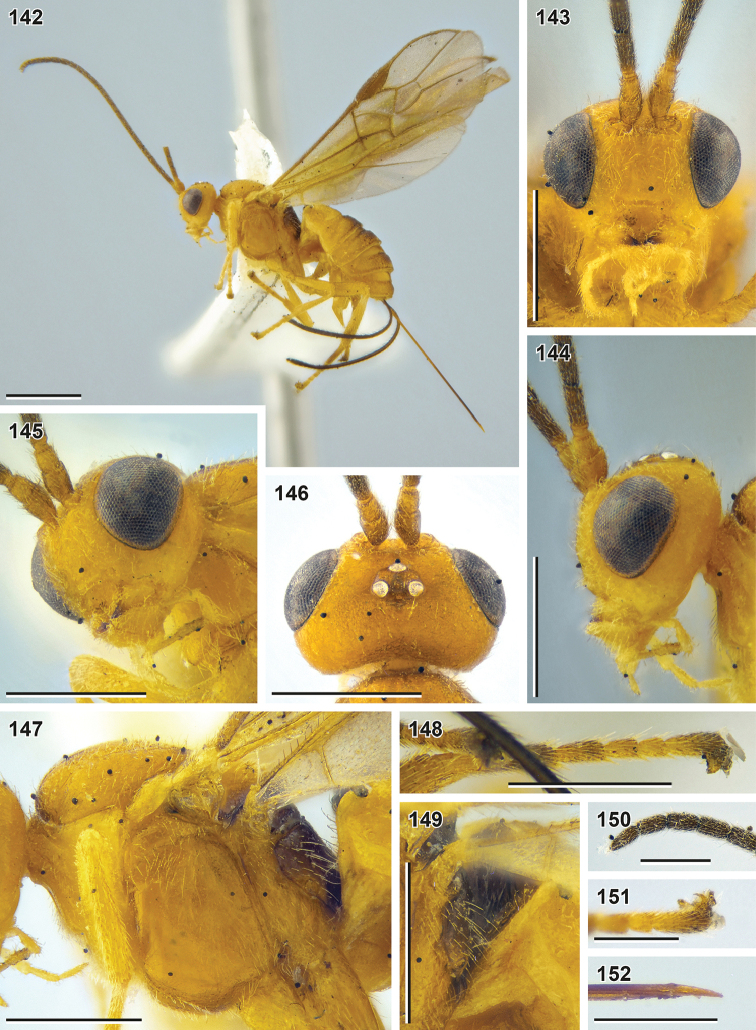
*Syntomernus scabrosus* sp. nov. (holotype, NIBR) **142** habitus, lateral view **143** head, front view **144** head, lateral view **145** head, ventrolateral view **146** head, dorsal view **147** mesosoma, lateral view **148** hind tarsus **149** first metasomal tergite **150** apex of antenna **151** apex of hind tarsus **152** apex of ovipositor. Scale bars: 1 mm (**142**); 0.5 mm (**143–149**); 0.25 mm (**150–152**).

##### Etymology.

The adjective *scabrosus* (Latin for scabrous) refers to the roughly sculptured metasoma of the species.

**Figures 153–156.**
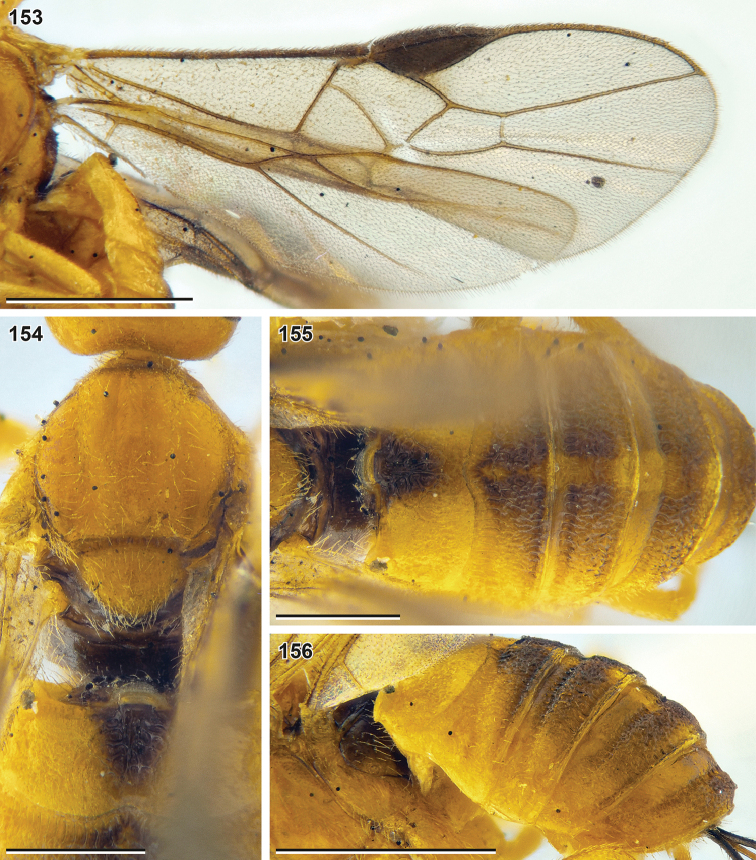
*Syntomernus scabrosus* sp. nov. (holotype, NIBR) **153** wings **154** mesosoma, dorsal view **155** metasoma, dorsal view **156** metasoma, dorsolateral view. Scale bars: 1 mm (**153, 156**); 0.5 mm (**154, 155**).

##### Description.

**Female.** Body length 3.1 mm; fore wing length 3.7 mm.

***Head*.** Width of head (dorsal view) 1.7× its median length. Transverse diameter of eye (dorsal view) 2.0× longer than temple. Eyes with sparse, short setae. OOL 2.4× Od; POL 1.2× Od; OOL 2.1× POL. Frons with deep mid-longitudinal groove. Longitudinal diameter of eye in lateral view 1.4× larger than its transverse diameter. Transverse diameter of eye (lateral view) 1.9× longer than minimum width of temple, hind margins of eye and temple parallel to broadened downwards. Face width 1.3× combined height of face and clypeus; 2.0× larger than width of hypoclypeal depression. Longitudinal diameter of eye 2.9× longer than malar space (front view); malar space 0.75× base of mandible. Malar suture absent. Width of hypoclypeal depression 1.3× larger than distance from depression to eye. Clypeus not separated from face by dorsal carina, flattened, with strongly protruding ventral rim, height of clypeus 0.32× width of hypoclypeal depression, clypeal sulcus smoothened. Maxillary palp longer than eye, but shorter than head.

***Antenna*** 0.87× as long as fore wing, with 26 antennomeres. First flagellomere 2.5× longer than its apical width, 1.1× longer than second flagellomere. Middle and penultimate flagellomeres 1.7× and 2.0× longer than wide, respectively. Apical flagellomere spiculate.

***Mesosoma*** 1.3× longer than its maximum height. Transverse pronotal sulcus deep and smooth. Notauli smooth, deep anteriorly, smoothened and not united posteriorly. Mesoscutum widely setose on notauli and anterolaterally, medially and latero-posteriorly widely glabrous. Scutellar sulcus crenulate. Mesepimeral sulcus smooth, mesopleural pit weak, furrow-like. Median area of metanotum (dorsal view) with incomplete median carina. Metapleural sulcus smooth. Mid-longitudinal keel developed in apical half of propodeum, simple and high. Propodeal spiracle vertical, located in middle of propodeum.

***Wings.*** Fore wing 1.2× longer than body. Pterostigma 2.6× longer than wide. Vein r arising from basal 0.38 of pterostigma. Vein 1-R1 1.6× longer than pterostigma. Marginal cell 8.3× longer than distance from its apex to apex of wing. Vein 3-SR 1.3× longer than vein r, 0.26× as long as vein SR1, 0.83× as long as vein 2-SR. Vein 1-M 0.67× vein 1-SR+M, 2.1× vein m-cu, 1.8× longer than vein cu-a. Vein 2-SR+M 0.21× as long as vein 2-SR, 0.48× as long as vein m-cu. Vein 1-CU1 (posterior margin of discal cell) 2.5× longer than vein cu-a. Vein cu-a interstitial. Vein 2-1A of hind wing very-very short; vein r-m strongly antefurcal.

***Legs.*** Fore tibia with longitudinal and transverse apical rows of thick setae. Hind femur 3.5× longer than wide. Hind tibia 1.4× longer than hind femur, with 2 thick setae subapically, its inner spur 0.4× as long as hind basitarsus. Hind tarsus 0.85× as long as hind tibia. Fifth segment (without pretarsus) of hind tarsus 0.6× as long as hind basitarsus and 1.2× longer than second segment. Claws with protruding blunt basal lobe.

***Metasoma*** 1.4× longer than mesosoma. Dorsolateral carinae of first metasomal tergite developed, dorsal carinae complete. Median area of first tergite separated by rugose furrow. First metasomal tergite with deep, crenulate mid-longitudinal impression. Second tergite medially 1.1× longer than third tergite. Basal width of second metasomal tergite 2.3× larger than its median length. Median area of second tergite weakly elevated, elongate triangular, separated by crenulate furrows, with complete sharp margin. Anterolateral areas of second tergite weakly elevated, with smoothened sculpture. Dorsolateral impressions of second tergite deep, s-shaped, crenulated. Spiracle of second metasomal tergite located behind middle of tergite. Suture between second and third tergites deep and wide, strongly curved and rugose. Apical margins of third to sixth tergites thick, with deep, crenulate transverse subapical grooves. Ovipositor sheath 2.2× longer than hind tibia and 0.6× as long as fore wing. Apex of ovipositor with developed nodus and ventral serration.

***Sculpture.*** Most of head and mesosoma smooth. Face weakly granulate, malar space granulate. First metasomal tergite laterally rugose, its median area weakly rugulose to rugose. Second–sixth tergites rugose.

***Colour.*** Head, scape, most of mesosoma and ground colour of legs and metasoma brownish yellow. Malar space, maxillary palps, pronotum laterally, tegulae, fore and most of middle leg, basal part of hind tibia yellow. Flagellum, apices of tarsi of legs, apex of hind tibia, hind basitarsus and third–sixth metasomal tergites brown. Metanotum, propodeum, first metasomal tergite and anteromedian patch on second metasomal tergite dark brown. Wing membrane weakly darkened; pterostigma and veins brown.

**Male.** Unknown.

##### Diagnosis.

The new species is easily recognisable by the entirely rugose metasoma, relatively short ovipositor, and enlarged fifth segment of the hind tarsus.

**Figures 157–166.**
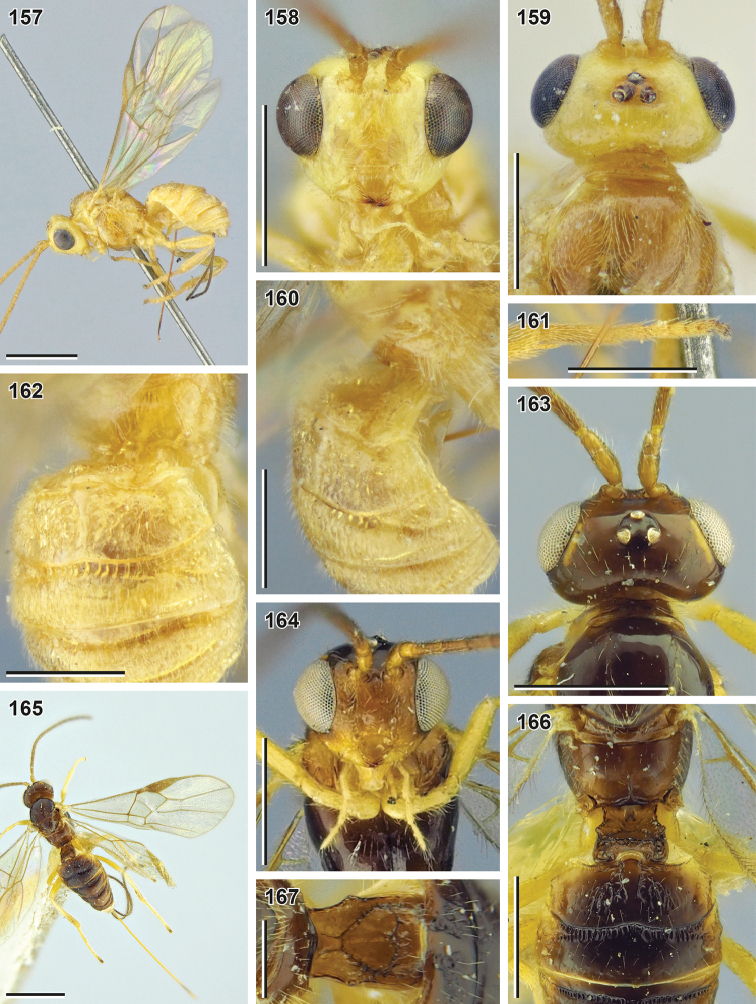
*Syntomernus pusillus* Enderlein, 1920 (**159–162** lectotype, MIIZ) and *S. sunosei* (Maeto, 1991) (**163–167** holotype of *Bracon flaccus* Papp, 1996, HNHM) **157** habitus, lateral view **158, 164** head, front view **159, 163** head, dorsal view **162, 160** propodeum and first–third metasomal tergites, dorsolateral view **161** hind tarsus **165** habitus, dorsal view **166** propodeum and first–third metasomal tergites, dorsal view **167** first metasomal tergite, dorsal view. Scale bars: 1 mm (**157, 165**); 0.5 mm (**158–164, 166**); 0.25 mm (**167**).

**Figures 168–178.**
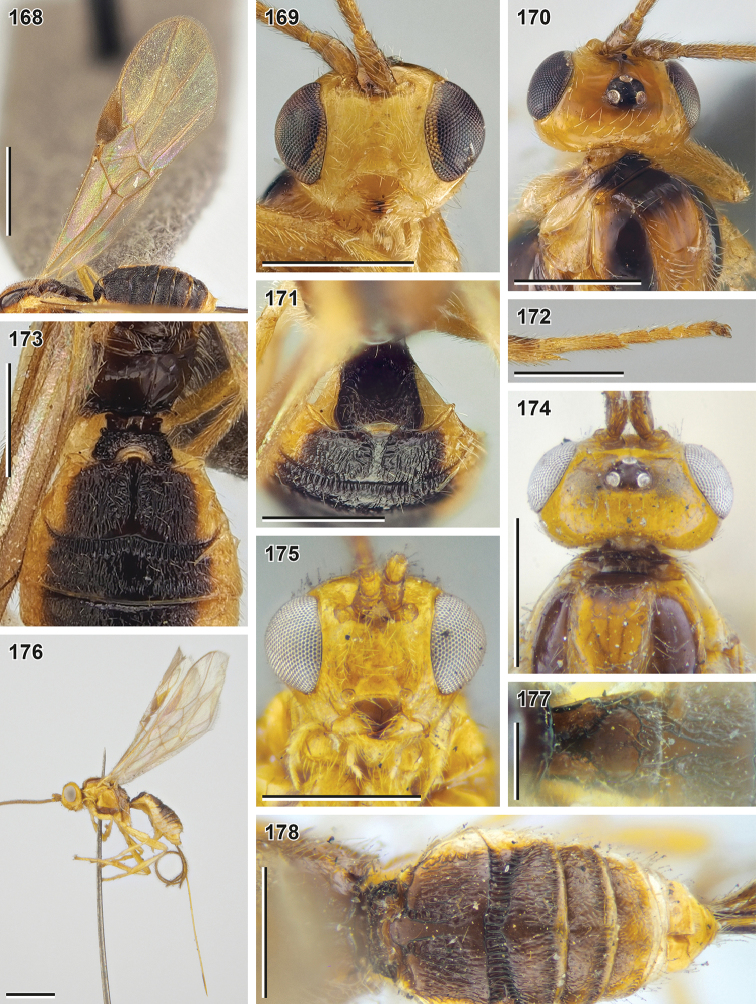
*Syntomernus tamabae* (Maeto, 1991) (**168–173** female, ZISP) and *S. asphondyliae* (Watanabe, 1940) (**174–178** paratype, EIHU) **168** fore wing **169, 175** head, front view **170, 174** head, dorsal view **173, 178** metasoma, dorsal view **172** hind tarsus **171, 177** first metasomal tergite, dorsal view **176** habitus, lateral view. Scale bars: 1 mm (**168, 176**); 0.5 mm (**169–175, 178**); 0.25 mm (**177**).

## Supplementary Material

XML Treatment for Bracon (Bracon) kimchanghyoi

XML Treatment for Bracon (Bracon) yeogisanensis

XML Treatment for Bracon (Habrobracon) allevatus

XML Treatment for Bracon (Osculobracon) perspicillatus

XML Treatment for Syntomernus

XML Treatment for Syntomernus flavus

XML Treatment for Syntomernus scabrosus
